# Optoplasmonic Effects in Highly Curved Surfaces for Catalysis, Photothermal Heating, and SERS

**DOI:** 10.1021/acsami.3c07880

**Published:** 2023-09-21

**Authors:** Jean-Francois Masson, Gregory Q. Wallace, Jérémie Asselin, Andrey Ten, Maryam Hojjat Jodaylami, Karen Faulds, Duncan Graham, John S. Biggins, Emilie Ringe

**Affiliations:** † Département de chimie, Quebec center for advanced materials, Regroupement québécois sur les matériaux de pointe, and Centre interdisciplinaire de recherche sur le cerveau et l’apprentissage, Université de Montréal, C.P. 6128 Succ. Centre-Ville, Montréal, QC Canada, H3C 3J7; ‡ Centre for Molecular Nanometrology, Department of Pure and Applied Chemistry, Technology and Innovation Centre, University of Strathclyde, 99 George Street, Glasgow G1 1RD, U.K.; § Department of Material Science and Metallurgy, University of Cambridge, 27 Charles Babbage Road, Cambridge, U.K. CB3 0FS; ∥ Department of Earth Science, University of Cambridge, Downing Street, Cambridge, U.K. CB2 3EQ; ⊥ Engineering Department, University of Cambridge, Trumpington Street, Cambridge, U.K. CB2 1PZ

**Keywords:** microspheres, fibers, Raman, SERS, catalysis, sensing, microscopy, photothermal heating

## Abstract

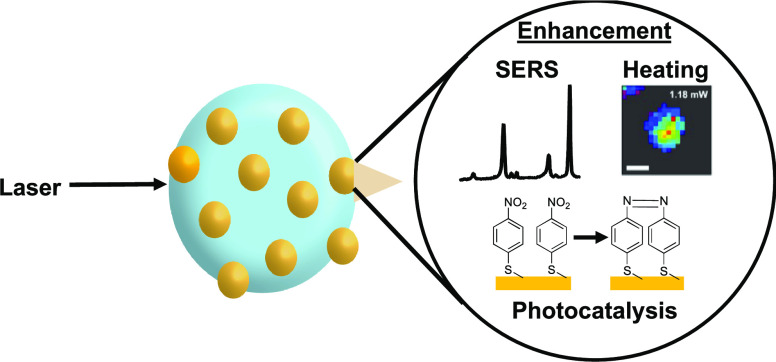

Surface curvature can be used to focus light and alter optical processes. Here, we show that curved surfaces (spheres, cylinders, and cones) with a radius of around 5 μm lead to maximal optoplasmonic properties including surface-enhanced Raman scattering (SERS), photocatalysis, and photothermal processes. Glass microspheres, microfibers, pulled fibers, and control flat substrates were functionalized with well-dispersed and dense arrays of 45 nm Au NP using polystyrene-*block*-poly-4-vinylpyridine (PS-*b*-P4VP) and chemically modified with 4-mercaptobenzoic acid (4-MBA, SERS reporter), 4-nitrobenzenethiol (4-NBT, reactive to plasmonic catalysis), or 4-fluorophenyl isocyanide (FPIC, photothermal reporter). The various curved substrates enhanced the plasmonic properties by focusing the light in a photonic nanojet and providing a directional antenna to increase the collection efficacy of SERS photons. The optoplasmonic effects led to an increase of up to 1 order of magnitude of the SERS response, up to 5 times the photocatalytic conversion of 4-NBT to 4,4′-dimercaptoazobenzene when the diameter of the curved surfaces was about 5 μm and a small increase in photothermal effects. Taken together, the results provide evidence that curvature enhances plasmonic properties and that its effect is maximal for spherical objects around a few micrometers in diameter, in agreement with a theoretical framework based on geometrical optics. These enhanced plasmonic effects and the stationary-phase-like plasmonic substrates pave the way to the next generation of sensors, plasmonic photocatalysts, and photothermal devices.

## Introduction

Nanoplasmonics exploit the interaction of light with nanostructures to enhance optical^1−3^ and physical^4,5^ effects occurring at the interface of the nanostructure and its environment to accomplish different tasks such as sensing (bio)molecules,^6−8^ catalyzing molecular reactions,^9,10^ or generating heat.^11^ When a surface plasmon resonance is excited on a nanostructure, the electric field is highly concentrated in a nanometric volume, leading to a large enhancement of spectroscopic signals (e.g., surface-enhanced Raman scattering (SERS)^3^ and luminescence^12,13^) and generating hot electrons capable of catalyzing reactions.^9,10,14^

A key research area in nanomaterials is the design of nanostructures that generate the large enhancement necessary to realize many nanoplasmonics applications.^15−17^ In addition to the design of structures with stronger electric field confinement or sharper resonances, one can also improve the performance of plasmonic processes by more efficiently coupling light into, and in some cases collecting light from, the nanostructure, giving rise to the field of optoplasmonics, where photonic elements are combined with plasmonic nanostructures. For example, this can be achieved with the use of optical fibers, where light/nanoparticle (NP) interaction occurs from the evanescent field, capable of generating a large absorbance from a relatively small number of particles.^18,19^ Yet, this strategy only provides better coupling and does not enhance the plasmonic properties.

Multiple interactions of light with nanostructures or further concentration of the light on the nanostructure can increase the probability of the plasmonic processes by allowing the photon multiple chances to excite the plasmon or by increasing the local field. The former can be achieved with optical microcavities,^20^ waveguided modes, or ring resonator modes in circular or spherical structures.^21,22^ In such structures, if constructive interference is achieved, the light races around the circumference and thus resides longer in the structure. These multiple passes or laps increase the probability of interaction with molecules^21,23,24^ and/or with plasmonic nanostructures.^25−27^

Further confinement of the light can be achieved with microlenses, with potential to improve light coupling in Raman and SERS.^28−30^ Focusing light with micrometric curved surfaces can lead to higher light power per unit area in nearby nanoparticles, especially critical in processes where the output is a nonlinear power function of the electric field such as SERS,^31^ the generation of hot electrons for catalysis,^32^ or the production of heat by photothermal effects.^33^ Better coupling of light with plasmonic nanostructures can also be achieved with waveguided modes in spherical microlenses.^34^ Finally, the higher collection efficiency of the scattered or reemitted light is another benefit of microlenses: their directional antenna effect concentrates the scattered or reemitted light in a narrower emission cone,^35−39^ making microlenses potentially advantageous for optoplasmonics.

A better understanding and expanded applications of microlensing in plasmonics will lead to better control over these processes and eventually to optimized applications. To date, in this context, Raman/SERS enhancement has been reported for chemical or Au NP monolayers,^28−30^ where microspheres played a passive role. A more interesting option, but only shown twice with submicron-sized silica beads,^40,41^ is an integrated optoplasmonic structure combining the optical element (microspheres) and the plasmonic nanostructures. For this type of structure, larger spheres may provide higher SERS enhancement,^28−30^ yet enhancement or size relationships remain to be shown on integrated plasmonic structures or for either catalysis or photothermal heating, two rising applications of plasmonics. Finally, the effect for plasmonically active structures was only shown in spherical optical components, but other shapes have similar optical properties,^42^ such as cylindrical (microfibers) or cones (tapered fibers), that could have interesting applications in smart textiles or sensing.

Here, we survey the optoplasmonic properties of different curved surfaces covered with well-dispersed and dense arrays of Au NPs by adapting a self-assembly method using block copolymer templating.^43−45^ Using polydisperse microspheres and microfibers, the influence of the diameter of the optoplasmonic structures on the plasmonically enhanced spectroscopic and catalytic properties is investigated. We confirm recent reports that suggested maximal Raman enhancement on microspheres of 5 μm,^46,47^ and show that this size relationship holds true for other types of curved surfaces. Importantly, we propose a theoretical framework to explain why a maximal response is observed at a specific diameter. We also extend the concept beyond SERS and show enhanced catalysis and photothermal effects in microspheres compared with flat surfaces. These results provide a comprehensive survey and understanding of the optoplasmonic properties in integrated microstructures, paving the way for new approaches to sensors, smart textiles, or catalysts.

## Experimental Section

### Synthesis of Au NPs

Au NPs were synthesized according to a previously reported seed-mediated growth method.^48^ In brief, seeds of approximately 12 nm were obtained by heating 80 mL of a 2.75 mM citrate buffer (3:1 trisodium citrate/citric acid) with 0.02 mM ethylenediamine tetraacetic acid (EDTA) to boil under vigorous stirring for 10 min then adding 20 mL of 0.8125 mM gold chloride dissolved in water. The reaction was stirred and boiled for 20 min to generate the seeds. These seeds were then further grown with successive additions of gold chloride and citrate. 2 mL of 34 mM trisodium citrate was added to 82.5 mL of water and boiled. To this, 2 mL of gold seeds were added, and after 1 min, 1.7 mL of 6.8 mM gold chloride was supplemented and refluxed for another 45 min. Successive additions of 2 mL of 34 mM trisodium citrate and 1.7 mL of 6.8 mM gold chloride were performed, once again boiling for 45 min between growth steps. Optical extinction spectra were measured between growth steps, and the process was stopped when approximately 45 nm spherical Au NPs were obtained. Size was measured using optical extinction spectroscopy with the method proposed by Haiss et al.^49^ and with scanning electron microscopy (SEM) (Figure S1). An optical density (O.D.) of approximately 2.5 was obtained for the as-synthesized Au NPs, which were stored at 4 °C until use. Immediately prior to their use, the Au NPs were centrifuged at 5000 rpm for 10 min, and half the supernatant was removed to concentrate the Au NPs to an O.D. of approximately 5, and a few drops of 100 mM HCl was added to adjust the pH around 5.

### Substrate Cleaning

Borosilicate rods of 1 mm diameter and 10 cm length were pulled on a P-2000 CO_2_ laser pipet puller (Sutter Instruments) similarly to a previously published protocol (Line 1–heat: 280, filament: 3, velocity: 15, delay: 145, pull: 20; Line 2–heat: 500, filament: 0, velocity: 15, delay: 128, pull: 200).^50^ The tip of the fiber was approximately 200 nm with these parameters, but one should note the parameters are instrument-dependent and optimization is needed to obtain similar pulled fibers on a different pipet puller. Various substrates including flat coverslips, glass microspheres (100 mg, 9–13 μm particle size, cat. no. 440345, Sigma-Aldrich), monodisperse borosilicate microspheres (20 mg, 5 μm, Duke Standards, cat. no. 9005, Thermo Fisher Scientific), pulled borosilicate rods (1 mm diameter solid borosilicate rods, Sutter Instruments) and microfibers (100 mg, 4 μm, cat. no 451040100, Acros Organics) were cleaned with approximately 5 mL of piranha (3:1 concentrated H_2_SO_4_:30%H_2_O_2_) for 40 min. *Caution, piranha is highly corrosive!* Surfaces were then rinsed with water until a neutral pH was reached. Macroscopic surfaces (coverslips and pulled fibers) were rinsed with ample water, while microfibers and glass beads suspension in piranha were diluted 10 times in water in 15 mL Falcon tubes, centrifuged at 5000 rpm for 2 min, and the supernatant was then discarded and rinsed with 10 mL of water once again. This process was repeated (typically 5 times) until a neutral pH was obtained for the supernatant.

### PS-*b*-P4VP Coating on the Substrates

The substrates were functionalized immediately after cleaning with polystyrene-poly-4-pyridine (PS-*b*-P4VP) block copolymer (MW: 41 kDa PS, 20 kDa P4VP; Polymer source, cat no. P11271-S4VP). A stock solution of the polymer was prepared by dissolving 1 mg/mL of PS-*b*-P4VP in tetrahydrofuran (THF) at 40 °C under gentle stirring. Dissolution typically took 30–40 min. The stock solution was kept for months at 4 °C. The stock solution was freshly diluted at 0.050 mg/mL in THF before further use. Coverslips and pulled fibers were first dried in air. The glass spheres and the microfibers were centrifuged at 5000 rpm for 2 min, and the supernatant was removed. The pellet was washed in tetrahydrofuran (THF) twice (centrifuged at 5000 rpm for 2 min between washes), and the pellet was left wet with a minimal volume of THF before adding PS-*b*-P4VP. The macroscopic substrates (coverslips and pulled fibers) were then manually dip-coated (immersion rate of approximately 1 mm/s), left for 3.5 min in the PS-*b*-P4VP solution, and manually withdrawn from the solution at a rate of approximately 1 mm/s. They were gently rinsed with THF and left to dry. The PS-P4VP solution (5 mL for 100 mg of substrate) was placed in the Falcon tube containing the glass spheres or the microfibers. After 3.5 min of immersion in PS-*b*-P4VP, the tubes were centrifuged at 5000 rpm for 2 min and the glass microspheres or microfibers were rinsed with THF 3 times (centrifuged at 5000 rpm for 2 min between washes).

### Coating Au NPs and a Chemical Monolayer on Substrates

After the last THF wash, the glass microspheres or microfibers were left wet with a minimal volume of THF and citrate-capped Au NPs (O.D. 5, pH ∼ 5) were added for 2 h. In the case of the coverslips, a drop of Au NPs was placed on the surface, and the tip of the pulled fibers was immersed in a drop of the Au NP suspension, also for 2 h. Once completed, the Au NP suspension was removed for the coverslips, and the pulled fibers were rinsed with an ample amount of water and left to dry in air. The glass microspheres and the microfibers were centrifuged at 2000 rpm for at least 2 min (sometimes 5 min was needed depending on the volume, *note the lower rpm to avoid the sedimentation of the free Au NPs*), rinsed three times with water if 4-mercaptobenzoic acid is used afterward or ethanol if 4-nitrobenzethiol (4-NBT) or 4-fluorophenyl isocyanide (FPIC) is to be coated on the substrate. Centrifugation at 2000 rpm for 2 min was done between washes. Finally, the substrates were coated for 2 h with 1 mM aqueous (4-MBA, dissolved in a minimal amount of ethanol, volume completed in water) or with 1 mM ethanolic solutions of 4-NBT. A stock 10 mM ethanolic FPIC solution was prepared. For functionalization, a final aqueous concentration of 10 μM was used, along with a functionalization time of 90 min. *Caution, FPIC is highly toxic. Manipulate under the fumehood!* The glass microspheres and the microfibers were centrifuged at 5000 rpm for 2 min and the substrates were rinsed three times with water or ethanol according to the solvent of the reaction. Between all of the steps, sonication and vortex shaking were used to resuspend the glass microspheres or microfibers. The suspension of glass microspheres or microfibers was then coated on a coverslip and left to dry. Substrates were then stored in the dark at room temperature until use.

### Optical and Electron Microscopy Characterization of the Au NP-Coated Substrates

Optical extinction spectra of the Au NP suspension were recorded on an Evolution 350 spectrophotometer (Thermo Fisher). Low-volume optical extinction spectra were measured with a BioTek Synergy neo2 multimode plate reader and a Take3 microvolume plate. For these experiments, microliters of the substrate suspension were pipetted in the microwells. Optical extinction spectra of the dry films of glass microspheres and microfibers were measured by drying a few drops of the substrate suspension on a 25 mm circular coverslip and placed at the focal point of a visible light microscope (40× objective), from which the light output was fiber coupled to a spectrophotometer (Princeton Instrument with an Andor camera).

SERS spectra were acquired with a Jobin-Yvon Horiba LabRam 300. The microscope was equipped with a 633 nm laser and an Olympus LMPlanFL 50X microscope objective (NA = 0.50). Unless otherwise specified, the spectra were accumulated for 1 s, and 5 spectra were averaged for each data point. Laser power was measured with a Thorlabs power meter (PM100A) equipped with an S120VC 200–1100 nm sensor. The SERS microscope was controlled by a LabSpec 6.0. For polydisperse substrates (glass microspheres and microfibers), sampling was done to obtain a similar number of data points to give relatively homogeneous weights of the diameter bins.

Photothermal heating experiments were performed using a custom-built optical setup equipped with a 785 nm laser at a laser power of ∼270 mW at the sample and a beam size of approximately 3 to 4 mm. For these measurements, 500 μL of the suspension was placed in a glass vial (volume of 1.75 mL) with a stir bar. The glass vial was then placed in a three-dimensional (3D)-printed holder positioned within the collimated beam path. The beam was aligned so that it passed through approximately the middle of the suspension. A thermocouple (Pico Technology) was placed in the suspension but out of the beam path. Stirring was maintained during photothermal irradiation to prevent the particles from settling out of the suspension over the duration of the experiment. Photothermal SERS spectra for the FPIC functionalized samples were performed with a Renishaw InVia Raman microscope controlled by Wire 4.4. The microscope was equipped with a 633 nm laser and a Leica N Plan EPI 50× microscope objective (NA = 0.75). A 1200 grooves/mm grating and an acquisition time of 1 s were used for all measurements. Laser power was measured with a Thorlabs power meter (PM100D) equipped with an S130C 400–1100 nm sensor. Mapping experiments of the Au NP-decorated silica spheres were performed with step sizes of 1 μm in the *x* and *y* axes. In all cases, photothermal SERS spectra and maps were processed by using a custom Python script.

Hyperspectral dark-field images were obtained with a Nikon Eclipse T*i* microscope equipped with a tungsten lamp, an Isoplane 320 spectrophotometer (Princeton Instruments), and a Princeton Instrument ProEM HS 1024 × 1024 EMCCD. Color images were acquired with a Thorlabs Kiralux camera. Glass microspheres and microfibers were measured with a 100× oil immersion objective (Nikon Plan Fluor, 0.16 mm working distance), and a S Plan Fluor ELWD 40X objective (NA = 0.6) was used for imaging the pulled fibers. The dark-field condenser (Nikon TI-DF dry condenser) had a NA of 0.8–0.95. In the case of parallel illumination, the condenser was removed, and a tungsten bulb (Thorlabs model QTH10/M) was placed about 10 cm from the substrate, providing a relatively collimated beam of light (no objective was used for illumination). The transmitted light was collected with an otherwise identical dark-field microscope. In all cases, data were processed with MatLab R2020a and with GraphPad Prism.

Electron microscopy images were collected with a FEI QEMSCAN 650F using 15 kV and a high vacuum to image the samples. Secondary electrons were imaged with an Everhart-Thornley detector (ETD) or a concentric backscatter detector (CBS) depending on the sample. Samples were placed on carbon tape to minimize charging, and no coating was deposited on the substrates.

## Results

The investigation of optoplasmonic effects on curved surfaces provides the opportunity to design better sensors and catalysts and improve photothermal effects. Here, we aim to unravel the details of this effect by studying the influence of differently curved surfaces and their diameters on plasmonic enhancement for different plasmonic processes and establish a theoretical framework to explain the optoplasmonic effects on curved surfaces. To observe optoplasmonic effects with highly curved surfaces, different surfaces (flat, microspheres, microfibers, and tapered pulled fibers) were coated with Au NPs and functionalized with different molecules to investigate the enhancement effect on sensing (using SERS as an example), on photocatalysis, and on photothermal effects.

### Characterization of the Optoplasmonic Properties of Highly Curved Microstructures

**Figure 1:**
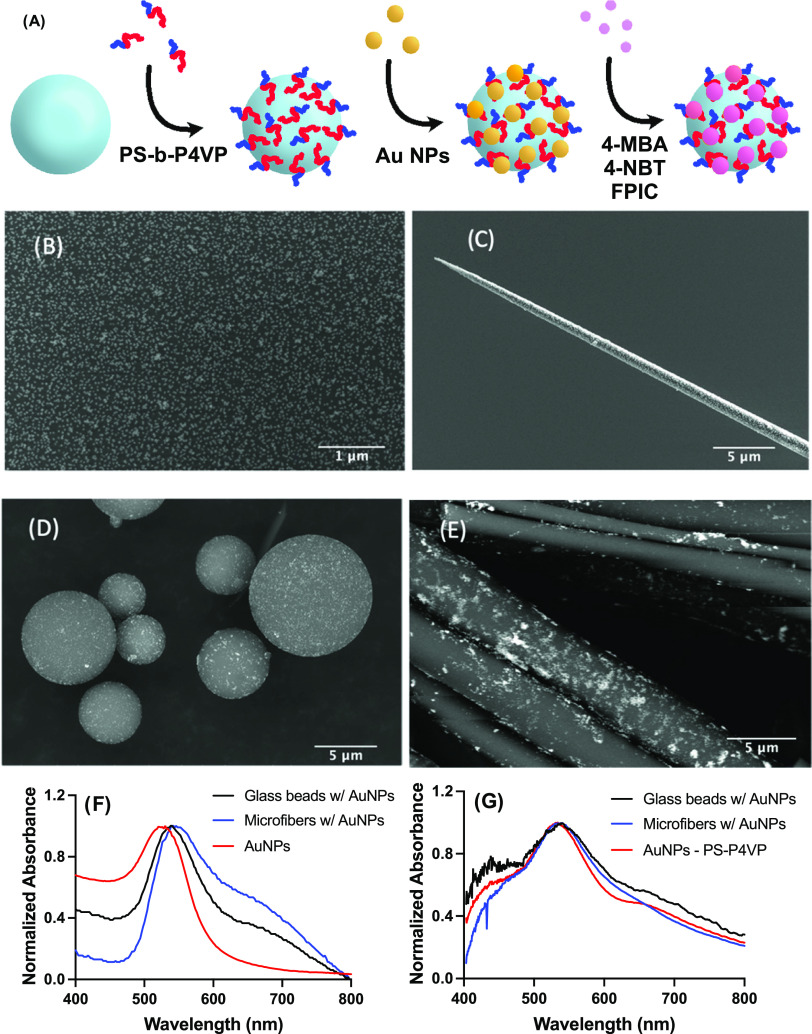
Decoration and characterization of the different highly curved substrates. (A) Scheme describing the synthesis process. Clean glass surfaces were functionalized with diluted (0.050 mM) PS-*b*-P4VP followed by the deposition of citrate-capped Au NP. The Au NP were functionalized with a monolayer of 4-mercaptobenzoic acid (4-MBA) for SERS, 4-nitrobenzenethiol (4-NBT) for photocatalysis, and 4-fluorophenyl isocyanide (FPIC) for photothermal studies. SEM images of the Au NP functionalized (B) flat coverslip, (C) pulled nanofibers, (D) polydisperse glass microspheres, and (E) microfibers. Optical extinction spectra for the aqueous Au NP suspension and aqueous suspension of (F) Au NP-coated substrates and (G) Au NP-coated substrates as a dry film.

To avoid aggregation of Au NPs, curved substrates were coated using a Au NP self-assembly method,^43−45^ providing reproducible surface coatings with minimal aggregation. Briefly, dip-coating a diluted PS-*b*-P4VP solution onto an acid-cleaned glass surface forms a brush polymer monolayer on the glass surface from the interaction of the P4VP block with the glass surface with the PS block extending in solution (Figure 1A). Citrate-capped Au NP suspension in a slightly acidic pH can interact with the surface-bound, positively charged P4VP and form well-dispersed, i.e., with minimal formation of aggregates, and dense arrays of Au NPs on flat substrates and pulled nanofibers as shown in Figure 1B,C and elsewhere.^44^ The particle density for the 45 nm Au NPs obtained here was estimated at 200 Au NPs/μm^2^ on flat substrates (Figure 1B), in agreement with our previous study,^45^ while pulled fibers were also coated with a well-dispersed array of Au NPs with the same density as on a flat substrate (Figure 1C), also in agreement with previous results.^43^

The PS-*b*-P4VP templating method was extended to other types of curved surfaces, namely, glass microspheres and microfibers, using a series of solution immersion steps without dip coating. Glass microspheres and microfibers with high size polydispersity (Figure S2) were used to evaluate the effect of diameter on optoplasmonic properties, while monodisperse glass beads of the optimal diameter were included to confirm optical properties (Figure S3). The glass microspheres were successfully coated with well-dispersed and dense arrays of Au NPs with some aggregation (Figure 1D), while Au NPs were deposited on microfibers less evenly than on other substrates (Figure 1E). This is attributed to the high degree of entanglement of the lengthy microfibers (Figure S2), preventing homogenization of the substrate in Au NP suspensions and uniform deposition of both PS-*b*-P4VP and Au NPs. While this uneven coating does not have an impact on the investigation of the effect of diameter, it will require consideration with respect to the SERS performance.

The different substrates were then measured with optical extinction spectroscopy to ensure comparable resonance wavelengths (Figure 1F). The Au NP suspension led to a plasmon resonance at 530 nm, slightly lower than those measured for aqueous suspensions of the glass microspheres (540 nm) and of the microfibers (543 nm). This is expected as Au NPs bound to a glass substrate experience a red shift due to the refractive index of glass being higher than that of water. The Au NPs deposited on microspheres and on microfibers showed noticeable absorbance in the 650–700 nm region, indicating aggregation. This aggregation was confirmed by SEM images (Figure 1B–E), which showed the formation of small Au NP aggregates on the substrates.

As the substrate will be used in the dry form in subsequent experiments, films of the different substrates were dried on glass coverslips to measure their optical properties. Au NPs deposited on PS-*b*-P4VP had a resonance at 533 nm, comparable to that of Au NPs on glass microspheres (538 nm) and microfibers (540 nm). The plasmon resonance was similar in all cases, as expected from the identical shapes and sizes of the Au NPs. The dry films also showed some aggregation for the different substrates, as all optical extinction spectra had a similar, yet rather small absorbance in the region indicating nanoparticle coupling (650–700 nm, Figure 1G). The extinction spectra from blank substrates (aqueous suspensions and dry films) showed no absorbance in the visible range (Figure S4), confirming that the optical properties arise solely from the Au NPs. These results show that the plasmonic properties of Au NPs on the different substrates are comparable and likely to have similar effects on the SERS, catalytic, and photothermal responses.

**Figure 2:**
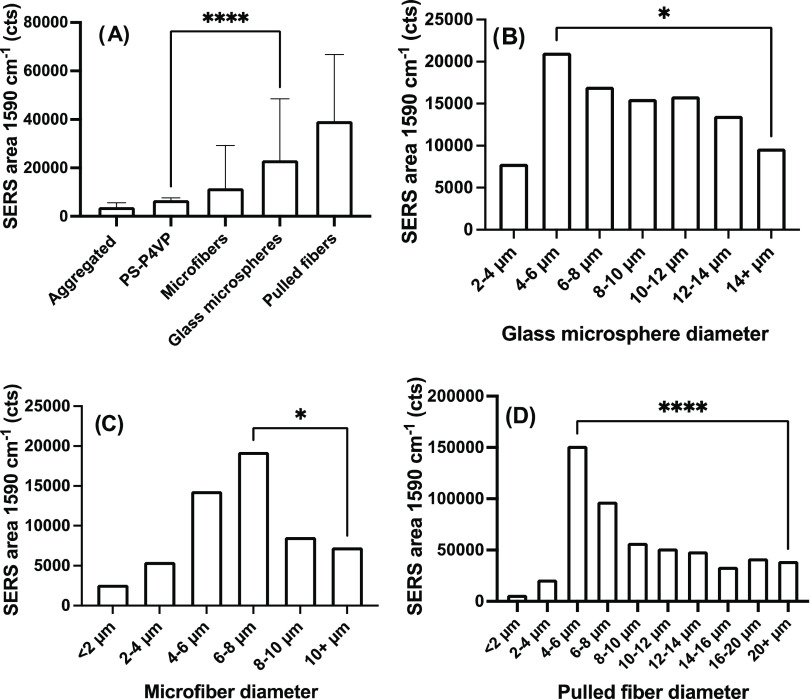
Influence of the substrate and its diameter on SERS properties. (A) Average area for the SERS for the peak at 1590 cm^–1^ of 4-MBA for the different substrates (**** *p* < 0.0001, *n* = 100 to 194 for all bins). The influence of the diameter of the different curved surfaces is shown with the median SERS area and is statistically significant between the optimal diameter and the largest diameters measured for (B) glass microspheres (* *p* < 0.05), (C) microfibers (* *p* < 0.05) and (D) pulled fibers (**** *p* < 0.0001). The number of data points per bin varied due to the randomness of the size for the polydisperse substrates and were for (B) glass microsphere *n* = 23 to 39 for all bins; (C) microfibers *n* = 5 for <2 μm; *n* = 14 to 88 for all other bins; (D) pulled fiber *n* = 6 for <2 μm; *n* = 13 to 74 for all other bins.

To analyze the effect of surface curvature on optoplasmonic properties, we looked for reproducible maximum enhancements of SERS, which is a reliable indicator of field enhancement.^51^ The SERS enhancement of Au NPs deposited on PS-*b*-P4VP favorably compares to that of otherwise identical Au NPs aggregated in solution and dried on a coverslip. Here, both substrates were coated with 4-MBA and SERS was measured with a laser excitation of 633 nm (Figure S5). The SERS response of the well-dispersed and dense arrays of Au NPs deposited with PS-*b*-P4VP led to average SERS intensities two times higher than for the dried aggregates, indicating that the polymer templating method improves SERS performance (Figure 2A). This result agrees with previous reports that dimers and trimers, such as those obtained with the polymer templating method, provide higher SERS than large aggregates of metallic NPs.^52^ In addition, the variability of the SERS signals on PS-*b*-P4VP-deposited Au NPs was significantly better at 14% coefficient of variation (CV, relative standard deviation) compared with 46% CV for the aggregated Au NPs. Polymer templating was used for the remainder of the study.

### Influence of Curvature on Optoplasmonic Effects

Microlensing effects using uncoated glass beads have previously been shown to improve the SERS response of flat substrates coated with Au NP.^46,47^ Here, glass beads were functionalized with Au NPs, integrating a microlens and plasmonic substrate amenable to homogeneous SERS assays or densely packed photocatalysts. In addition to spherical microstructures, similar experiments were performed on microfibers (cylindrical) and pulled fibers (conical) to evaluate the generality of the curvature effects on SERS enhancement.

SERS enhancements ranging from 3 to 10 times were obtained on the different curved surfaces in comparison to the aggregated Au NPs or up to 5 times in relation to the flat substrate with Au NPs templated with PS-*b*-P4VP (Figure 2A). This is a similar performance gain to that previously reported for a Au NP substrate covered with glass microspheres,^46^ showing that integrating the plasmonic substrate to the curved surface has a similar effect on the SERS performance. The results in Figure 2A also demonstrate that SERS is enhanced compared to a flat surface irrespective of the type of curved surfaces (spherical, cylindrical, and conical).

The polydispersity of the glass microspheres and microfibers (Figure S2) and the tapered cone of the pulled fibers (Figure 1C) allowed researchers to investigate the effect of diameter on the SERS response. In all cases, the integrated area of the 4-MBA peak at 1590 cm^–1^ was maximal for diameters of approximately 5 μm, consistent with prior observations that enhancement is maximal at that diameter,^46,47^ whereas both lower and higher diameters led to lower SERS enhancements (Figures 2B–D). In the case of microfibers, the optimal diameter was slightly higher at 6–8 μm but nonetheless consistent with the other curved substrates. Quantifying the enhancement more accurately was possible with monodisperse glass beads of 5.3 μm (Supporting Information, Text Section 1), for which an enhancement of the SERS intensity by a factor of 6 was obtained in comparison to a flat surface (Figure S6). It should be noted that the microspheres technically have twice the number of particles as a flat surface since the laser interacts with both sides of the microspheres. Finally, since the dependence of SERS intensity on size was relatively similar for the different types of curved surfaces, glass microspheres will be used as a model substrate in the next experiments, but similar results would be expected with microfibers or pulled fibers.

### Influence of Surface Curvature on Plasmonic Catalysis

**Figure 3:**
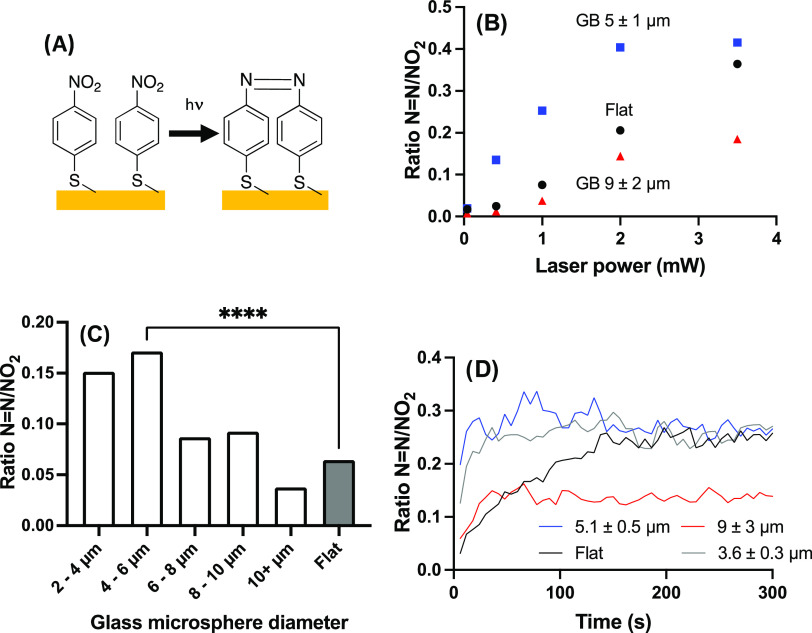
Photocatalysis of 4-nitrobenzenethiol (4-NBT) on gold nanoparticles under laser irradiation at 633 nm. (A) Molecular structures of the reagent (4-NBT) and the product of the photoreaction (4,4′-dimercaptoazobenzene). (B) Laser power dependence on the extent of initial photoconversion (*n* = 10 for each data point, the size label in the legend represents the mean diameter and one standard deviation). (C) Relative SERS intensity of the N=N peak to NO_2_ peak as a function of the diameter of glass microspheres. *n* = 43 to 64 for the different diameters, while *n* = 100 for the flat surface, ****p < 0.0001. (D) Kinetic trace of the photocatalytic reaction rate at 1 mW laser power (*n* = 6 or 7 for each trace, the size label in the legend represents the mean diameter and one standard deviation). Data points were acquired every 6 s for 300 s.

Plasmon-driven photocatalysis is a vibrant field where the electric field enhancement provided by the excitation of plasmon with light modifies chemical reactions and catalytic processes on plasmonic substrates.^9,10,53^ Here, Au NPs deposited on a flat surface or on glass microspheres were functionalized with 4-NBT. This molecule undergoes dimerization when excited with laser light, a process that is laser wavelength- and power-dependent, leading to the reduction of the NO_2_ group and the formation of 4,4′-dimercaptoazobenzene (4,4′-DMAB) (Figure 3A), providing a probe for photocatalytic processes.^53^

The SERS spectra at the low laser power of 0.041 mW (beam diameter of 2.5 μm, hence 0.84 kW/cm^2^) displayed the characteristic Raman scattering peaks of 4-NBT at 858, 1079, 1111, 1339, and 1574 cm^–1^ (Figure S7A), in agreement with the literature.^54^ Increasing the laser power to 1.0 mW (20 kW/cm^2^) revealed significant changes in the SERS spectra (new peaks at 1022, 1145, 1187, 1394, 1445, and 1482 cm^–1^, Figure S7A) in accordance with the vibrational peaks reported for 4,4′-DMAB^54,55^ proving that photocatalysis of 4-NBT to 4,4′-DMAB is obtained in the current conditions. The peaks of interest for this reaction are the 1339 cm^–1^ peak associated with the NO_2_ group of 4-NBT which is converted to –N=N– in 4,4′-DMAB, corresponding to the 1445 cm^–1^ peak. The intensity ratio of these two peaks provides a relative measure of the photoconversion under different conditions and will be used to compare the photocatalytic performance of the flat surface and the glass microspheres.

Laser power impacts photocatalytic processes: the photoconversion was almost linear with laser power for the flat surface and larger glass microspheres (diameter ≥6 μm), while the smaller glass microspheres reached a plateau at 2 mW laser power (Figure 3B), likely due to saturation of the photoconversion. Photoconversion remains incomplete under these conditions, as the NO_2_ peak of 4-NBT remains visible in the SERS spectra, albeit at a lower relative intensity. The higher –N=N–/NO_2_ ratio with NP-coated glass microspheres indicated several fold enhancement of the photocatalytic conversion due to the optoplasmonic effects of the curved surface of the glass microspheres. This enhancement was maximal for a laser power of 0.41 mW (8.4 kW/cm^2^) and reduced for higher laser power, as the reaction reached a plateau within the 5 s integration time for both the flat substrate and the glass microspheres coated with Au NPs (Figure S7B). The low laser power of 0.041 mW (0.84 kW/cm^2^) did not induce significant photocatalysis either on Au NP-coated glass microspheres or on a flat substrate.

As shown in Figure 3C, the photocatalysis yield is dependent on the size of the glass microspheres. Experiments were conducted at the single sphere level on approximately 50 spheres coated with Au NPs. Maximal photocatalytic conversion of 4-NBT to 4,4′-DMAB was obtained with 4–6 μm glass microspheres, similarly to the maximal SERS enhancement with glass microspheres of the same size. Smaller glass microspheres of 2–4 μm had marginally poorer performance than the 4–6 μm glass microspheres although the photocatalytic conversion ratio was not statistically different. The photocatalysis performance rapidly declined for glass microspheres larger than 6 μm (statistically different, *p* < 0.01) compared to that of the 4–6 μm glass microspheres, and even further declined with ≥10 μm glass microspheres or flat substrates (*p* < 0.0001). Photoconversion of 4-NBT was also greater on the Au NPs on monodisperse glass microspheres of 5.3 μm in diameter; for these substrates, the ratio of the –N=N–/NO_2_ SERS intensity was 3.7 times larger than for the flat surface (Figure S7C).

To survey the kinetics of this photocatalysis, spectra were collected at 1.0 mW laser power for 5 min at 6 s intervals (5 s integration +1 s file transfer and start of new acquisition). A clear increase in the catalytic rate was observed for smaller glass microspheres compared with a flat substrate. The reaction reached a plateau within the first 30 s of illumination under these conditions for spheres smaller than 6 μm (Figure 3D). The rate was slightly faster for the 4–6 μm glass microspheres compared to the smaller glass microspheres, in agreement with Figure 3C. Saturation of the photocatalysis conversion was observed at the same –N=N–/NO_2_ ratio for the smaller glass microspheres and the flat surface (albeit at a lower rate). In the case of the larger glass microspheres, the initial photoconversion rate was similar to that of the flat surface before saturating at a lower photocatalytic conversion ratio. Taken together, these results confirm that photocatalysis is enhanced when Au NPs are supported on glass microspheres of approximately 5 μm, consistent with the SERS results, providing evidence that other plasmonic phenomena are improved with highly curved surfaces.

### Influence of Surface Curvature on Plasmonically Induced Photothermal Heating

Plasmonic NPs generate heat following optical excitation,^56^ which can lead to relatively high surface temperatures. Photothermal effects can be observed from a change in bulk temperature in the presence of Au NPs,^57^ while the surface temperature of plasmonic substrates can be estimated from the change in the Raman bands of a probe molecule, such as phenyl isocyanides including 4-fluorophenyl isocyanide (FPIC).^58,59^ As the phenyl isocyanide experiences an increase in temperature, its orientation at the surface of the metal changes, resulting in a blue shift of the cyanide Raman peak.

Bulk temperature measurements were performed under 785 nm laser illumination in a glass vial equipped with a thermocouple. The change in laser wavelength, compared to previous experiments, was due to instrument availability. A suspension of the glass microspheres coated with Au NPs led to a temperature increase of nearly 20 °C (Figure S8). While this temperature rise is significant in comparison to Au NPs in suspension (about 2 °C), most of the gain in temperature seems to arise from the bare glass microspheres (about 10 °C). The dramatic difference in the photothermal properties of the Au NPs-coated microspheres and the bare glass microspheres is the result of the strong scattering properties of the glass microspheres. Given that the temperature probe is placed directly into the solution, it is likely that photons scattered by the glass microspheres can interact with the temperature probe, leading to an observed increase in temperature, also observed elsewhere.^60^ If the probe is placed directly into the path of the beam, the temperature increase is both sharper and higher than what is shown in Figure S8. The additional heating caused by adding the Au NPs onto the glass microspheres is likely the result of cooperative optical effects. One scenario is that the presence of the Au NPs on the surface of the glass microspheres alters the absorption and scattering properties of the base components leading to a change in the extinction cross section. Alternatively, the increase in scattering caused by the glass microspheres could allow for more photons to interact with Au NPs either on the same sphere or on adjacent spheres. This then results in additional heating caused by the absorption of the scattered light by the Au NPs. To better evaluate the photothermal properties of the microspheres coated with Au NP, surface temperature measurements were performed as opposed to bulk temperature measurements.

**Figure 4:**
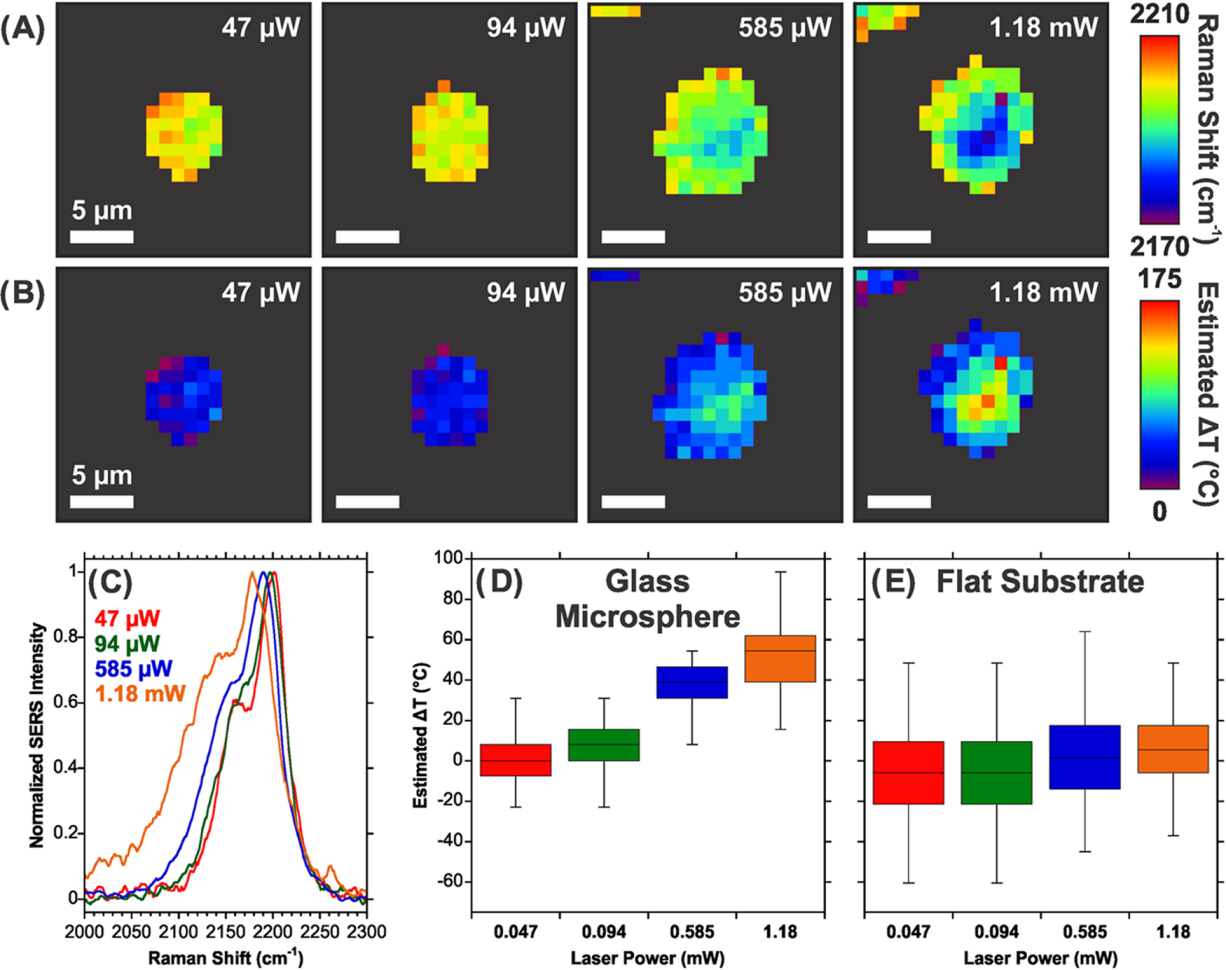
Photothermal effect on 4-fluorophenyl isocyanide (FPIC) on glass microspheres coated with Au NP. (A) SERS imaging of the CN band at different laser powers showing a stronger photothermal effect at the center of the microsphere with larger laser powers and (B) the corresponding temperature estimates. (C) SERS spectra at the different laser powers and temperature changes based on ensemble measurements on (D) glass microspheres and (E) Au NP on a flat substrate. *n* = 35 for each data point in (D) and (E). For (D) and (E), the average Raman shift at 47 μW was used to determine the initial temperature. A response of 0.2 °C/cm^–1^ was used to estimate the temperatures.

Surface temperatures were estimated from the shift in the CN vibration (∼2200 cm^–1^) for FPIC under laser illumination and measured with SERS. Imaging single glass spheres led to strong Raman shifts, especially near the center of the microspheres coated with Au NPs (Figure 4A,B), similar to SERS measurements of 4-MBA on the coated microspheres (Figure S9), providing strong evidence of microlensing. The position of the Raman peaks scale with laser power for Au NPs on glass microspheres (Figure 4C,D), in agreement with prior results on Au NPs.^58^ Applying the calibration from refs (58,59), where an ∼0.2 cm^–1^ blue shift per °C increase in temperature was observed, temperature changes in excess of 100 °C are estimated at the highest laser power (Figure 4B). Comparing the Raman peak positions of the Au NPs on the glass microsphere and the control Au NPs on a flat substrate were compared, larger rises in estimated temperature were observed as the laser power increased for the glass microspheres (Figure 4D,E).

In addition to the shift in the CN vibrational energy, noticeable changes in the SERS spectra of FPIC were observed as the laser power increased (Figure S10). In some instances, new peaks appeared, but consistently, a broad band between 1400 and 1700 cm^–1^ rose, superimposed on the existing vibrational modes. Like the results with 4-NBT (Figure S11), these changes have been previously associated with hot charge carrier-induced changes,^61^ which can cause photocarbonization of FPIC, residual citrate, and/or the block copolymer. Another possible cause of photocarbonization is the higher surface temperatures observed on the plasmonic substrates.

The conversion of 4-NBT to 4,4′-DMAB is also believed to be driven by hot charge carriers,^62^ but photothermal effects also occur during plasmonic photocatalysis.^63^ For the glass microspheres coated with Au NPs, at low to moderate laser powers, 4-NBT selectively converts to DMAB as evidenced by an increase in the –N=N–/NO_2_ ratio (Figure 3B). Yet, photocarbonization occurs more readily as the laser power increases, and this effect becomes apparent at a much lower laser power on the microspheres than on a flat substrate (Figures S10–S12). The further increase in the surface temperature may play a role in improving the photocatalytic activity, but it is also important to recognize that additional processes will occur. The previously determined ideal laser power of 0.41 mW (8.4 kW/cm^2^) for photocatalysis appears to be near the threshold for generating carbon species at the surface of the Au NP (Figures S11, S12) indicating that higher temperatures are generated and perhaps can contribute to photocatalysis.

### Theoretical Framework for an Optimal Diameter

The results showed that the enhancement of photocatalysis, photothermal heat generation, and SERS intensity of different curved surfaces are maximal at a diameter of approximately 5 μm. An experimental model was previously proposed for the SERS enhancement based on the photonic nanojet, directional emission, waveguided modes or resonances, and enhancement of the plasmonic effects.^38^ The plasmonic enhancement can also be modeled by numerical simulations, as reported elsewhere for collimated illumination,^46^ but this approach has limitations due to the multilength scale features of the substrate and the illumination geometry obtained with a microscope objective. The main concerns are the difficulty of modeling focused rays and the computational power required to model “large” microspheres coated with an array of much smaller Au NPs. While informative, the previous models predicted a field enhancement from the microspheres but did not consider either the angular dependence of the illumination in plasmonically enhanced spectroscopies (*e.g.*, SERS) or the curvature radius of the surface. Therefore, neither of the previous approaches predicted our observed size-dependent results.

#### Optical Paths in Microlenses

**Figure 5:**
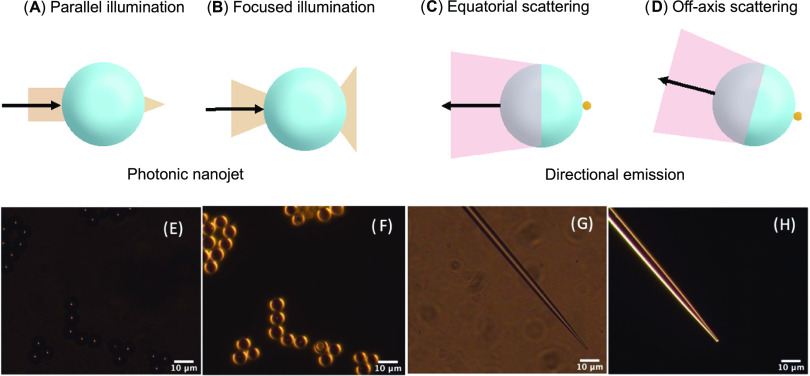
Scheme representing the illumination path for (A) parallel rays or (B) focused rays with a beam width defined by the microscope objective on a glass microsphere (experimentally: 2.5 μm diameter). Optical path of the backscattered Raman photons for (C) a particle placed at the equator and (D) at the edge of the illuminated area for focused rays. The characterization of the standard 5.3 μm glass microspheres and pulled fibers coated with Au NP provide experimental evidence of the model in (A) and (B). The optical images were acquired for (E) glass microspheres under collimated and (F) dark-field illumination, as well as for (G) pulled fibers under collimated and (H) dark-field illumination.

The trajectory of light rays in glass microspheres depends on the illumination orientation, and this is the dominant factor in explaining the diameter dependency of the optoplasmonic properties. Taking the boundaries of possible trajectories to model the effect of a ball lens, the simplest one considers collimated rays impinging the curved surface parallel to the equator. In this current model, a two-dimensional (2D) circular surface is considered because it models the cross section of a sphere (glass microspheres), a cone (pulled fibers), or a cylinder (microfibers). These rays will experience focusing analogous to a ball lens (Figure 5A) and prior models only considered these rays in predicting the optical properties of microspheres. Given that most results above were obtained with a Raman microscope equipped with a 50× objective NA 0.5, we also needed to model the rays impinging the surface with a range of angles from 0 to 30° (Figure 5B). These rays will be refracted by the glass microsphere in a different trajectory than collimated rays. The net result is a focal point inside the ball lens and rays that then diverge at the opposite pole of the microsphere, describing a larger illumination solid angle than the parallel, collimated rays. The solid angle described by these light rays focused on the microsphere will be dependent on the size of the focused beam impinging on the microsphere, on the diameter of the bead, and the incident angle (more details are provided in Supporting Information, Text Section 2).

Experimental evidence supports the angular dependence of the optical properties in relation to light excitation, confirming the hypothesis raised in Figure 5A,B. Taking the two extreme cases, collimated light (incidence angle = 0°) and dark-field illumination (incidence angles from 53 to 72° - NA 0.80–0.95) led to different optical effects for both the glass microspheres (Figure 5D,E) and pulled fibers (Figure 5G,H), in agreement with the proposed model (Figure 5A,B). Parallel illumination with collimated light created a photonic nanojet, extensively studied previously,^64,65^ visible from the lensing effect observed as the bright spot at the center of the glass microsphere (Figure 5E) or a line of intense light across the long axis of the pulled fiber (Figure 5G). The photonic nanojet was further revealed by taking a cross section of the light intensity (Figure S13A).

Dark-field illumination favored the illumination on the outer edge of the spheres (Figure 5F) or the pulled fiber (Figure 5H), which is also evident from the cross section light intensity (Figure S13B). In that case, ring resonator modes were observed (Figure S13C,D), which were more evident in the microspheres but also present in the pulled fiber. The modes observed at different wavelengths agreed well with theoretical predictions for *l* between 31 and 39 for a 5.3 μm sphere, where *l* is an integer corresponding to the number of periods traveling within the circumference of the sphere. However, while present, these resonances were not strong. Yet, the excitation of plasmon resonances was observed with parallel or angled illumination (Figure S14). Therefore, a simple approximation can be made that the plasmonic enhancement (SERS and photocatalysis) should be related to the photonic nanojet intensity and, to a lesser extent, by the waveguided mode due to the highly scattering nature of the Au NPs on the microsphere limiting their propagation in the microsphere.

**Figure 6:**
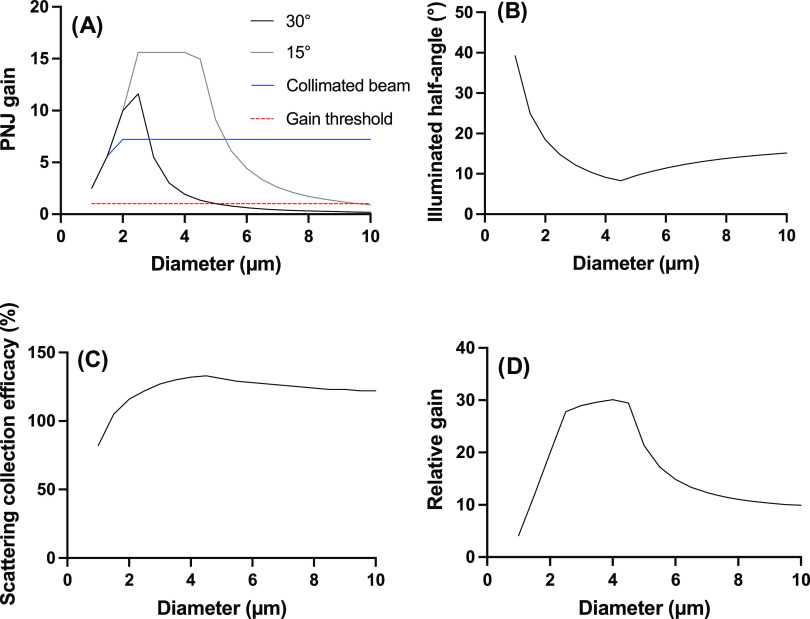
Predictions from the theoretical framework using diffraction-limited geometrical optics. (A) Theoretical gain for a glass microsphere illuminated with collimated rays (blue) or focused rays impinging the sphere at 15° (gray) or 30° (black) for a laser beam of 2.5 μm diameter. The dashed red line demarks the threshold where a gain in illumination intensity is obtained. (B) Illuminated half-angle for the face of the microsphere opposite to the incident laser beam. Diameters <4.5 μm are in the diffraction-limited regime, while diameters ≥4.5 μm are limited by the divergence of the laser beam within the glass microspheres from the focused laser beam at 15°. (C) Estimated collection efficacy of the backscattered Raman photons considering the combination of a Au NP on the equator and another off-axis at the edge of the solid angle described by the focused illumination. (D) Relative gain predicted from the combination of the PNJ effect for collimated beams and at 15° with the collection efficacy of the directional antenna. Values in graphs (A–D) are relative to the conditions modeled. They do not reflect the anticipated gain for experimental conditions. The values for each trace in the panels were calculated at 0.5 μm diameter intervals.

As the angular dependence on the optical properties is clearly shown above, a simple geometrical optic framework is proposed here to describe the ray path and the solid angle described by the focused rays (Figure S15). The model considers the impact of diameter on the photonic nanojet, the illuminated area, and the collection efficiency of the scattered light. The photonic nanojet occurs when light rays are focused by a curved surface to an area smaller than that of the incident beam (Figure 5A). The light intensity will therefore be larger on the opposite face of the glass microsphere and enhance plasmonic processes. This is, of course, only possible for nearly collimated rays and a beam diameter larger than the diffraction limit (633 nm in the current case). A photonic nanojet gain (PNJ_gain_) can be described by the ratio of the incident beam area on the microspheres to the one described on the opposite pole of the microsphere or, equivalently, the ratio of the square of the radius of the incident beam (y) to the radius of the beam at the opposite pole (*x*).
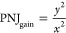
1In the experiments reported here, the microscope focused the beam to a diameter of approximately 2.5 μm, and the rays had a distribution of incident angles from 0 to 30° given the NA (0.5) of the objective. Vertical rays (0°) are focused by the microspheres using ball lens geometrical optics, where the PNJ_gain_ is low for very small microspheres, and then approximately constant with diameters above 2 μm (Figure 6A). The maximum light concentration by the photonic nanojet is observed when the beam size is equal to the microsphere diameter, in agreement with previous experimental evidence for a collimated beam.^66^ Focused rays show a different diameter dependency (Figure 6A), where a maximum PNJ_gain_ is expected for diameters between 2.5 and 4.5 μm for an incidence angle of 15°. Higher incidence angles (i.e., 30°) will lead to lower PNJ_gain_, maximized at smaller diameters, due to the more divergent trajectory of these light rays.

Using the model described in the SI, the PNJ_gain_ should increase indefinitely with smaller spheres, but geometrical optics eventually breaks down due to the diffraction limit. Hence, for an incidence angle of 15° and a beam diameter of 2.5 μm, the light rays will be constrained to the diffraction limit for beads smaller than 5 μm, explaining the plateau observed in Figure 6A. In addition, one also needs to consider that the incident beam is larger than microspheres smaller than 2.5 μm; thus, only a fraction of the light will impinge on the microspheres, explaining the decrease in PNJ_gain_ for microspheres smaller than 2.5 μm. As such, the PNJ_gain_ is maximal for microspheres of about 2.5 to 4.5 μm.

The scattered rays on the opposite pole of the microspheres can be refracted back to the microscope objective by the glass microsphere; the directional antenna phenomenon (Figure 5C). This can lead to a gain in the collection efficiency of backscattered rays, which is especially critical for SERS enhancement. It was previously reported that the backscattered rays will be refracted at a maximum angle of 14° by a microsphere,^46^ limited by refraction in a glass microsphere. Hence, rays backscattered up to the critical angle (43.3° for glass in air) will be captured by the microscope objective (NA = 0.50, 30° cone angle) as these rays will be refracted with a maximum angle of 14° (Figure S16), a 1.44 times gain compared to the collection angle of 30° of backscattered photons with the same microscope objective and in the absence of the glass microsphere.

The construction above is true only for rays backscattered from the pole of the microsphere. As the refracted light by the microsphere illuminates a solid angle, the backscattered rays from the illuminated Au NP that are located off the optical axis described by the microscope objective and the microsphere will be refracted at an angle rotated from that optical axis (Figure 5D) and thus, fewer photons will be captured by the microscope objective depending on the solid cone angle illuminated on the microsphere (Figure 5B). That solid cone angle is diameter-dependent; it decreases with diameter up to the diffraction limit of light, which for the current parameters modeled (2.5 μm beam diameter and 15° incidence angle) will be minimal at 4.5 μm diameter (Figure 6B). As the beam is diffraction-limited below that diameter, the solid cone angle increases rapidly at smaller diameters leading to worse collection efficacy of the backscattered rays. Hence, it is predicted that the backscattered SERS photons will have a maximum collection efficiency close to 4.5 μm (Figure 6C) due to the geometrical optics constraint of the directional antenna.

While the theoretical framework based on geometrical optics explains the diameter dependency of the optoplasmonic properties, it has some limitations. The diameter was optically measured, but the incident angle is estimated from the fact that the laser beam impinging on the microscope objective is relatively collimated, and its waist is smaller than the microscope objective. This will lead to a lower NA of the objective for the laser beam. Extreme cases were modeled based on these assumptions, but rays in a focused beam have a broad distribution of incidence angles and trajectories. These various possibilities are not accounted for by the model, but the extreme cases should provide a reasonable estimate of the solid angle described by refracted light in the microsphere. The same argument applies to the directional antenna phenomenon, where only two extreme cases (equatorial Au NPs and off-axis Au NPs) at the edge of the illuminated solid angle are modeled. As such, the PNJ_gain_ and scattering collection efficacy are not absolute values but provide a relative indication of the trends. However, gains predicted here are on the same order of magnitude as those measured in ref (30), where the authors measured that the photonic nanojet improved SERS by about 1 order of magnitude and that the directional antenna led to a 4- to 5-fold improvement of the response. This compares well with the 3 to 10 times enhancement that we observed. The diffraction limit had to be added to the model, as pure geometrical optics calculations would lead to the impossible scenario of light confinement below the diffraction limit. Finally, the model does not provide an indication of the intensity distribution in the refracted rays, which would have an effect on the plasmonic processes occurring in the microspheres.

The model is based on a circular cross section of the different types of curved surfaces that provide qualitative agreement with spherical, cylindrical, or conical surfaces. While for microspheres the results discussed above can be directly extended to the third dimension by projecting the results into the polar coordinates of a sphere, the results would be slightly different for conical or cylindrical surfaces. Considering the photonic nanojet, a cylindrical or conical surface would have a more diffuse linear focus (see Figure 5G for conically pulled fibers). However, the size dependence is expected to be similar, even though the lensing effects would be a convolution of the different diameters of the tapered pulled fiber illuminated by the focused beam since the small taper angle of 13° results in a relatively narrow diameter distribution within the illuminated spot. As such, the model serves as a good qualitative representation of the expected SERS response for different types of curved surfaces.

Despite some limitations, the predictions from the photonic nanojet model at 15° and for collimated rays with the directional antenna explain the trends observed experimentally. The SERS collection efficiency is based on the combination of photonic nanojet, illumination half-angle, and directional antenna phenomena. Thus, the relative ratio of the SERS intensity (Figure 2B) is close to that of the theoretical model considering all of these phenomena (Figure 6D), where the SERS intensity has a slight decrease for glass microspheres above 5 μm. A minor difference was observed in the relative ratio of plasmonic photocatalysis on glass microspheres of different sizes (Figure 3C) compared to the SERS intensity profile for glass microspheres of the same size (Figure 2B). This is due to the fact that the plasmonic photocatalysis mainly depends on the gain in the photonic nanojet, which increases the local intensity of the electric field, and not on the collection efficiency of the scattered photons from the illumination angle or the collection efficiency from the directional antenna. Thus, the relative plasmonic photocatalysis on microspheres of different diameters (Figure 3C) closely matches the gain in the photonic nanojet (Figure 6A), where the photocatalytic ratio decreases more rapidly for microspheres above 6 μm. A similar agreement with Figure 6A is expected for the photothermal properties with different diameters.

Thus, we conclude that the observation of the strong diameter dependency on the SERS, catalysis, and photothermal phenomena requires that the beam diameter exceeds the diffraction limit, that it approximately matches the optimal size of the microspheres, and that the incidence angle of the beam should not be too large to minimize the solid angle described by the refracted beam on the microsphere. In summary, we showed that geometrical optics eventually breaks down for microlenses on the order of a few microns; thus, glass microspheres of about 2.5 to 4.5 μm are predicted to be optimal for optoplasmonics, in general agreement with the experimental evidence that microspheres of approximately 5 μm led to maximal enhancements.

## Conclusions

Optical effects in various curved surfaces improved plasmonic phenomena such as SERS, photocatalysis, and photothermal effects. The optoplasmonic properties of Au NP-coated substrates revealed the excitation of surface plasmons and different optical modes under varying illumination paths. As a result of the shape of the curved surface, SERS was improved by at least up to an order of magnitude in glass microspheres, microfibers, or pulled fibers in comparison to otherwise identically prepared flat Au NP-coated substrates when microstructures of 5 μm diameter were illuminated with a laser beam. Photocatalytic conversion of 4-NBT to 4,4′-DMAB was up to 5 times more efficient in glass microspheres than on flat substrates, and a maximal gain was also observed for glass microspheres of about 5 μm. Photothermal data suggested that the microspheres contributed to a small increase in temperature. The optimal diameter is rationalized as an effect of the angled illumination of the laser light on curved surfaces and of the beam diameter being larger than the diffraction limit of light improving the photonic nanojet and the directional antenna phenomena. As such, we conclude that geometrical optics breaks down for curved surfaces with diameters of a few micrometers, restricted by the diffraction limit of light. Taken together, these results revealed the interesting optoplasmonic properties of highly curved surfaces and their potential for the design of next-generation sensors, photocatalysts, or photothermal heat generators.

## Supporting Information Available

Optoplasmonic properties of 5.3 μm glass microspheres and the predictive model; optical and electron microscopy images, extinction and Raman spectra of the substrates, comparative graphs of the SERS, and photocatalytic and photothermal data (PDF)
