# Supplementary material for: Optoplasmonic Effects in Highly Curved Surfaces for Catalysis, Photothermal Heating, and SERS
Source: ACS Appl Mater Interfaces. 2023 Sep 21;15(39):46181–94. doi: 10.1021/acsami.3c07880 (PMC10561152; doi:10.1021/acsami.3c07880)
Supplement: Supplementary file 1 — am3c07880_si_001.pdf [file am3c07880_si_001.pdf]

Supporting Information for:

Optoplasmonic Effects in Highly Curved Surfaces for Catalysis, Photothermal Heating and SERS

Jean-Francois Masson[1*], Jérémie Asselin[2,3], Andrey Ten[2,3], Gregory Q. Wallace[1], Maryam Hojjat Jodaylami[1], Karen Faulds[4], Duncan Graham[4], John S. Biggins[5], and Emilie Ringe[2,3]

[1] *Département de chimie, Quebec center for advanced materials, Regroupement québécois sur les matériaux de pointe, and Centre interdisciplinaire de recherche sur le cerveau et l'apprentissage, Université de Montréal, C.P. 6128 Succ. Centre-Ville, Montréal, QC, Canada, H3C 3J7*

[2] *Department of Material Science and Metallurgy, University of Cambridge, 27 Charles Babbage Road, Cambridge, UK CB3 0FS*

[3] *Department of Earth Science, University of Cambridge, Downing Street, Cambridge, UK CB2 3EQ*

[4] *Centre for Molecular Nanometrology, Department of Pure and Applied Chemistry, Technology and Innovation Centre, University of Strathclyde, 99 George Street, Glasgow, G1 1RD, UK.*

[5] *Engineering Department, University of Cambridge, Trumpington Street. Cambridge, UK CB2 1PZ*

Corresponding authors information: *jf.masson@umontreal.ca*, *er407@cam.ac.uk*

Supplementary text:

1. Optoplasmonic properties of monodispersed 5.3 µm glass microspheres

SERS and photocatalytic performance of the curved microstructure was shown to be optimal for diameters around 5 µm. Glass microspheres of 5.3 µm were then tested as they are expected to hold significant advantages for nanoplasmonic applications. The monodispersity of the substrate leads to lower variance in SERS; where the variance of the monodisperse was half of the polydisperse one. The variance of the glass microspheres could be further decreased if the center of the bead could be probed accurately, as the maximum SERS response coincides with the illumination of the center of the bead (Figure S8).

To better characterize the optoplasmonic properties at that length scale, glass microspheres with a standard size of 5.3 ± 0.4 µm were functionalized identically to the previous experiments. SEM images confirm the monodispersity of the glass microspheres and functionalization with a well-disperse and dense array of Au NPs (Figure S3). Normalizing the spectra collected for the Au NP-coated monodisperse glass microspheres to the one of bare substrates revealed the excitation of plasmon resonances. In the case of parallel illumination with collimated light, an increase in the scattering was observed at the plasmon resonance (around 600 nm) for both the microspheres (Figure S10A) and the pulled fiber (Figure S10C). This is in opposition to dark-field illumination, which led to an increase in absorption near 520 nm for the microsphere (Figure S10B), caused by the different light path in the curved surface. This effect was not observed in pulled fibers (Figure S10D), probably due to an even lower Q factor of the resonator given the different diameters illuminated in this conical structure.

2. Predictive model for the diameter influence on the plasmonic properties:

    a. Photonic nanojet : Collimated rays

The model for collimated rays is based on ball lens equations [1], where the microspheres act as a perfect lens. To calculate the illuminated area on the opposite side of the microsphere, one must calculate the effective focal length (EFL), the back focal length (BFL) and the numerical aperture (NA) of the lens considering the finite size of the beam at the focal plane of the microscope objective.

The EFL and BFL are functions of the diameter (*D*) of the microsphere and its refractive index (*n*).

$$EFL = \frac{nD}{4(n-1)} \qquad \text{(S1)}$$

$$BFL = EFL - \frac{D}{2} \qquad \text{(S2)}$$

The NA is also a function of the microsphere diameter, but also of the beam diameter (*d*), the refractive index, which will describe a cone of light with an angle (θ_{BL}).

$$NA = n \sin \theta_{BL} = \frac{1}{\sqrt{1+4(\frac{nD}{4d(n-1)})^2}} \qquad \text{(S3)}$$

From these equations, the radius of the illuminated area can be calculated, which was then used for the calculation of the photonic nanojet gain (PNJ_{gain}) with equation 1 in the main text.

$$r_{illuminated} = BFL \ \tan(\theta_{BL}) \qquad \text{(S4)}$$

Table S1. Parameters for collimated rays on microspheres of different diameters with a beam diameter of 2.5 µm and glass of *n* = 1.457

D (µm)	*r (µm)*	*BFL (µm)*	*EFL (µm)*	*NA*	θ_{BFL}	$r_{illuminated}$ *(µm)*	*PNJ gain*
1.0	0.5	0.80	0.30	0.53*	32.1	0.317**	2.5
1.5	0.75	1.20	0.45	0.53*	32.1	0.317**	5.6
2.0	1	1.59	0.59	0.53*	32.1	0.373	7.2
2.5	1.25	1.99	0.74	0.53	32.1	0.466	7.2
3.0	1.5	2.39	0.89	0.46	27.6	0.466	7.2
3.5	1.75	2.79	1.04	0.41	24.1	0.466	7.2
4.0	2	3.19	1.19	0.37	21.4	0.466	7.2
4.5	2.25	3.59	1.34	0.33	19.2	0.466	7.2
5.0	2.5	3.99	1.49	0.30	17.4	0.466	7.2
5.5	2.75	4.38	1.63	0.27	15.9	0.466	7.2
6.0	3	4.78	1.78	0.25	14.6	0.466	7.2
6.5	3.25	5.18	1.93	0.23	13.6	0.466	7.2
7.0	3.5	5.58	2.08	0.22	12.6	0.466	7.2
7.5	3.75	5.98	2.23	0.20	11.8	0.466	7.2
8.0	4	6.38	2.38	0.19	11.1	0.466	7.2
8.5	4.25	6.77	2.52	0.18	10.5	0.466	7.2
9.0	4.5	7.17	2.67	0.17	9.9	0.466	7.2
9.5	4.75	7.57	2.82	0.16	9.4	0.466	7.2
10.0	5	7.97	2.97	0.15	8.9	0.466	7.2
10.5	5.25	8.37	3.12	0.15	8.5	0.466	7.2
11.0	5.5	8.77	3.27	0.14	8.1	0.466	7.2
11.5	5.75	9.17	3.42	0.14	7.8	0.466	7.2
12.0	6	9.56	3.56	0.13	7.4	0.466	7.2
12.5	6.25	9.96	3.71	0.12	7.2	0.466	7.2
13.0	6.5	10.36	3.86	0.12	6.9	0.466	7.2
13.5	6.75	10.76	4.01	0.12	6.6	0.466	7.2
14.0	7	11.16	4.16	0.11	6.4	0.466	7.2
14.5	7.25	11.56	4.31	0.11	6.2	0.466	7.2
15.0	7.5	11.96	4.46	0.10	6.0	0.466	7.2
15.5	7.75	12.35	4.60	0.10	5.8	0.466	7.2
16.0	8	12.75	4.75	0.10	5.6	0.466	7.2
16.5	8.25	13.15	4.90	0.09	5.4	0.466	7.2
17.0	8.5	13.55	5.05	0.09	5.3	0.466	7.2
17.5	8.75	13.95	5.20	0.09	5.1	0.466	7.2
18.0	9	14.35	5.35	0.09	5.0	0.466	7.2
18.5	9.25	14.75	5.50	0.08	4.8	0.466	7.2
19.0	9.5	15.14	5.64	0.08	4.7	0.466	7.2
19.5	9.75	15.54	5.79	0.08	4.6	0.466	7.2
20.0	10	15.94	5.94	0.08	4.5	0.466	7.2

* The microsphere is smaller than the beam diameter. Thus, the diameter of the microsphere is also the beam diameter.

** The calculated $r_{illuminated}$ is smaller than the diffraction limited radius of light (0.3165 µm for 633 nm laser), thus the diffraction limit is used for the calculation.

b. Photonic nanojet : Focused rays

The microscope objective focuses rays on a sample such as the curved surfaces of microspheres, which curvature will change refraction of light in the substrate. The incident light describes a cone on the samples, whose diameter and angle are defined by the optics in the microscope objective (Figure S10). In the case of the SERS and photocatalysis experiments, a 633 nm laser was focused on the samples using a 50X objective with a NA = 0.5. As the NA is 0.5, the diffraction limit of the light will also be 633 nm. We experimentally measured the diameter of the laser beam at 2.5 µm, or a radius (y) of 1.25 µm. The angle of the laser rays with respect to the incident plane normal (ϕ) can be comprised between 0 and 30°. We simulated two cases, at 30°, the most extreme case, and 15°, the probable case experimentally. This incident ray then intersects the glass sphere with a refraction normal (α) that is a function of the laser beam radius (y) and the sphere radius (r) (equation S5):

$$\alpha = \sin^{-1}(y/r) \qquad \text{(S5)}$$

The incidence angle (θ) is the difference between the incident plane normal (ϕ) and the refraction normal (α):

$$\theta = \alpha - \phi \qquad \text{(S6)}$$

The angle of the refracted ray can be calculated from Snell's law, where n_1 is air and thus approximately 1 and omitted in the equation:

$$\gamma = \sin^{-1}(\sin\theta / n_2) \qquad \text{(S7)}$$

The next steps use a series of right angle triangular decompositions of the beam path to calculate the distance (*x*) from the center of the microsphere where the ray will intersect the surface, describing a solid angle cone (β):

$$\delta = \alpha - \gamma \quad \text{(S8)}$$

$$f = r \cos \alpha \quad \text{(S9)}$$

$$a = f / \cos \delta \quad \text{(S10)}$$

$$\omega = 90 - \delta \quad \text{(S11)}$$

$$\sigma = 90 - \omega \quad \text{(S12)}$$

$$c = y - a \sin \delta \quad \text{(S13)}$$

$$b = r \sin (180 - \gamma - \omega) / (\sin \omega) \quad \text{(S14)}$$

$$y = (a+b) \sin d \quad \text{(S15)}$$

$$x = y - b \quad \text{(S16)}$$

$$\beta = \sin^{-1}(x/r) \quad \text{(S17)}$$

Table S2. Parameters for focused rays on microspheres of different diameters with a beam diameter of 2.5 µm and glass of *n* = 1.457

		Incidence angle 15°			***Incidence angle 30°***		
D (µm)	*r (µm)*	$r_{illuminated}$ *(µm)*	*β (°)*	*PNJ gain*	$r_{illuminated}$ *(µm)*	*β (°)*	*PNJ gain*
1.0	0.5	0.317*	39.3	2.5	0.317*	39.3	2.5
1.5	0.75	0.317*	25.0	5.6	0.317*	25.0	5.6
2.0	1	0.317*	18.5	10.0	0.317*	18.5	10.0
2.5	1.25	0.317**	14.7	15.6	0.367	17.1	11.6
3.0	1.5	0.317**	12.2	15.6	0.534	20.8	5.5
3.5	1.75	0.317**	10.4	15.6	0.721	24.3	3.0
4.0	2	0.317**	9.1	15.6	0.901	26.8	1.9
4.5	2.25	0.323	8.3	15.0	1.08	28.6	1.3
5.0	2.5	0.414	9.5	9.1	1.25	30.0	1.0
5.5	2.75	0.505	10.6	6.1	1.42	31.1	0.8
6.0	3	0.595	11.4	4.4	1.59	32.0	0.6
6.5	3.25	0.686	12.2	3.3	1.76	32.7	0.5
7.0	3.5	0.775	12.8	2.6	1.92	33.3	0.4
7.5	3.75	0.865	13.3	2.1	2.09	33.9	0.4
8.0	4	0.954	13.8	1.7	2.26	34.3	0.3
8.5	4.25	1.04	14.2	1.4	2.42	34.7	0.3
9.0	4.5	1.13	14.6	1.2	2.58	35.1	0.2
9.5	4.75	1.22	14.9	1.0	2.75	35.4	0.2
10.0	5	1.31	15.2	0.9	2.91	35.6	0.2
10.5	5.25	1.40	15.5	0.8	3.08	35.9	0.2
11.0	5.5	1.49	15.7	0.7	3.24	36.1	0.15
11.5	5.75	1.58	15.9	0.6	3.40	36.3	0.13
12.0	6	1.66	16.1	0.6	3.57	36.5	0.12
12.5	6.25	1.75	16.3	0.5	3.73	36.6	0.11
13.0	6.5	1.84	16.5	0.5	3.89	36.8	0.10
13.5	6.75	1.93	16.6	0.4	4.05	36.9	0.09
14.0	7	2.02	16.8	0.4	4.22	37.0	0.09
14.5	7.25	2.11	16.9	0.4	4.38	37.2	0.08
15.0	7.5	2.19	17.0	0.3	4.54	37.3	0.08
15.5	7.75	2.28	17.1	0.3	4.70	37.4	0.07
16.0	8	2.37	17.2	0.3	4.87	37.5	0.07
16.5	8.25	2.46	17.3	0.3	5.03	37.6	0.06
17.0	8.5	2.55	17.4	0.2	5.19	37.6	0.06
17.5	8.75	2.63	17.5	0.2	5.35	37.7	0.05
18.0	9	2.72	17.6	0.2	5.51	37.8	0.05
18.5	9.25	2.81	17.7	0.2	5.68	37.9	0.05
19.0	9.5	2.90	17.8	0.2	5.84	37.9	0.05
19.5	9.75	2.99	17.8	0.2	6.00	38.0	0.04
20.0	10	3.07	17.9	0.2	6.16	38.0	0.04

* The microsphere is smaller than the beam diameter and the calculated $r_{illuminated}$ is smaller than the diffraction limited radius of light (0.3165 µm for 633 nm laser), thus the diffraction limit is used for the calculation.

** The calculated $r_{illuminated}$ is smaller than the diffraction limited radius of light (0.3165 µm for 633 nm laser), thus the diffraction limit is used for the calculation.

c. Directional antenna

The Raman photons are scattered isotropically. Among all Raman photons, only backscattered rays can be collected by the Raman epi-microscope. In addition, only the photons refracted within the collection angle of the microscope objective (30°) will be detected. Photons entering total internal reflection should not be detected, albeit there is a chance they could be scattered towards the microscope objective following one or multiple total internal reflections. For the following calculation, this probability is deemed much smaller than refraction and thus total internal reflected rays are not considered.

The power of the collected Raman photons can be estimated from the solid angle detected by the microscope objective. In absence of microspheres, the solid angle (χ) is limited by the numerical aperture of the microscope objective (NA = 0.5 or χ_{wo_sphere} = 30°). For a particle located on the equator on the opposite side of the incident rays, backscattered rays within a cone of ± 43.3° will be refracted, corresponding to the critical angle of the glass|air interface. The refracted rays are calculated with Snell's law, where the incident medium is glass (RI = 1.457) and air constitutes the second medium. The far-field radiation angle (χ_{FF}) is a relation of the scattering angle (χ_{scat}) and the refracted angle (χ_{ref}), as shown in Table S3 and Figure S8.

$$\chi_{FF} = 2\,\chi_{scat} - \chi_{ref} \qquad \text{(S18)}$$

This angle is independent of the sphere diameter and as shown in Figure S8, all far-field radiation angles (43.3°, or 86.6° for the solid angle) are comprised in the 30° collection angle (or 60° for the solid angle) of the microscope objective. Thus, the directional antenna effect increases the collection efficacy of the SERS photon by 1.44 times:

$$\text{Enhancement} = 60°/86.6° \qquad \text{(S19)}$$

Table S3. Far-field radiation angle for different scattering angles in glass microspheres of n = 1.457

χ_{scat} (°)	χ_{ref} (°)	χ_{FF} (°)	χ_{cat} (°)	χ_{ref}(°)	χ_{FF}(°)
1	1.5	0.5	21	31.5	10.5
2	2.9	1.1	22	33.1	10.9
3	4.4	1.6	23	34.7	11.3
4	5.8	2.2	24	36.3	11.7
5	7.3	2.7	25	38.0	12.0
6	8.8	3.2	26	39.7	12.3
7	10.2	3.8	27	41.4	12.6
8	11.7	4.3	28	43.2	12.8
9	13.2	4.8	29	44.9	13.1
10	14.7	5.3	30	46.8	13.2
11	16.1	5.9	31	48.6	13.4
12	17.6	6.4	32	50.5	13.5
13	19.1	6.9	33	52.5	13.5
14	20.6	7.4	34	54.6	13.4
15	22.2	7.8	35	56.7	13.3
16	23.7	8.3	36	58.9	13.1
17	25.2	8.8	37	61.3	12.7
18	26.8	9.2	38	63.8	12.2
19	28.3	9.7	39	66.5	11.5
20	29.9	10.1	40	69.5	10.5
			41	72.9	9.1
			42	77.1	6.9
			43	83.6	2.4

The collection efficiency of the Raman photons differs if a particle emits at the edge of the illuminated cone in the case of a focused beam due to the rotation angle of the refracted scattered photons, which points away from the microscope objective (Figure 4D). The collected cone is dictated by a rotation angle corresponding to the cone angle β and is limited by the microsphere's pole, thus a maximum half-angle of 45°, or a difference of 1.7° in comparison to the equatorial scenario.

$$\text{Collected\_angle} = 86.6° - \beta + 1.7° \quad \text{(S20)}$$

The collection efficacy for Au NPs at the edge of the illuminated cone is given by:

$$\text{Collection efficacy\_edge} = \text{Collected angle} / 86.6° \qquad \text{(S21)}$$

The estimated collection efficacy is the product of the two collection efficacies for the equatorial rays and the ones and the edge of the illuminated cone. This provides an estimate, as photon are scattered by Au NPs located in the entire illuminated cone.

Table S4. Estimation of the collection efficacy for the directional antenna

D (µm)	*r (µm)*	β (°)	*Collected angle (°)*	*Collection efficacy -edge (%)*	*Collection efficacy – equatorial (%)*	*Estimated collection efficacy (%)*
1.0	0.5	39.3*	49	56.6	144	82
1.5	0.75	25.0*	63.3	73.1	144	105
2.0	1	18.5*	69.8	80.6	144	116
2.5	1.25	14.7**	73.6	85.0	144	122
3.0	1.5	12.2**	76.1	87.9	144	127
3.5	1.75	10.4**	77.9	90.0	144	130
4.0	2	9.1**	79.2	91.5	144	132
4.5	2.25	8.3	80	92.4	144	133
5.0	2.5	9.5	78.8	91.0	144	131
5.5	2.75	10.6	77.7	89.7	144	129
6.0	3	11.4	76.9	88.8	144	128
6.5	3.25	12.2	76.1	87.9	144	127
7.0	3.5	12.8	75.5	87.2	144	126
7.5	3.75	13.3	75	86.6	144	125
8.0	4	13.8	74.5	86.0	144	124
8.5	4.25	14.2	74.1	85.6	144	123
9.0	4.5	14.6	73.7	85.1	144	123
9.5	4.75	14.9	73.4	84.8	144	122
10.0	5	15.2	73.1	84.4	144	122
10.5	5.25	15.5	72.8	84.1	144	121
11.0	5.5	15.7	72.6	83.8	144	121
11.5	5.75	15.9	72.4	83.6	144	120
12.0	6	16.1	72.2	83.4	144	120
12.5	6.25	16.3	72	83.1	144	120
13.0	6.5	16.5	71.8	82.9	144	119
13.5	6.75	16.6	71.7	82.8	144	119
14.0	7	16.8	71.5	82.6	144	119
14.5	7.25	16.9	71.4	82.4	144	119
15.0	7.5	17.0	71.3	82.3	144	119
15.5	7.75	17.1	71.2	82.2	144	118
16.0	8	17.2	71.1	82.1	144	118
16.5	8.25	17.3	71	82.0	144	118
17.0	8.5	17.4	70.9	81.9	144	118
17.5	8.75	17.5	70.8	81.8	144	118
18.0	9	17.6	70.7	81.6	144	118
18.5	9.25	17.7	70.6	81.5	144	117
19.0	9.5	17.8	70.5	81.4	144	117
19.5	9.75	17.8	70.5	81.4	144	117
20.0	10	17.9	70.4	81.3	144	117

* The microsphere is smaller than the beam diameter and the calculated $r_{illuminated}$ is smaller than the diffraction limited radius of light (0.3165 µm for 633 nm laser), thus the diffraction limit is used for the calculation.

** The calculated $r_{illuminated}$ is smaller than the diffraction limited radius of light (0.3165 µm for 633 nm laser), thus the diffraction limit is used for the calculation.

Supplementary Figures:

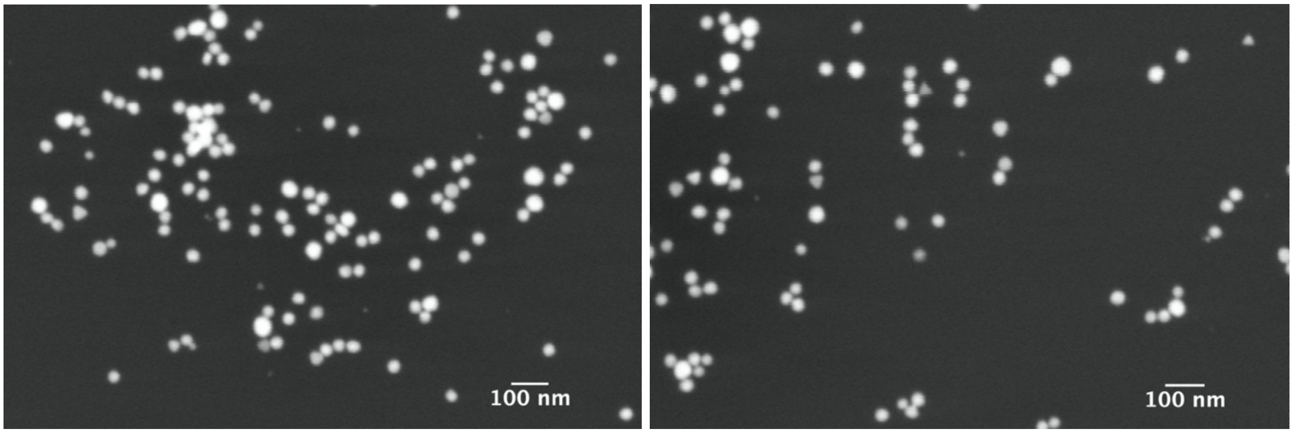

Figure S1. SEM images (backscattered electrons) of the as-synthesized Au NPs.

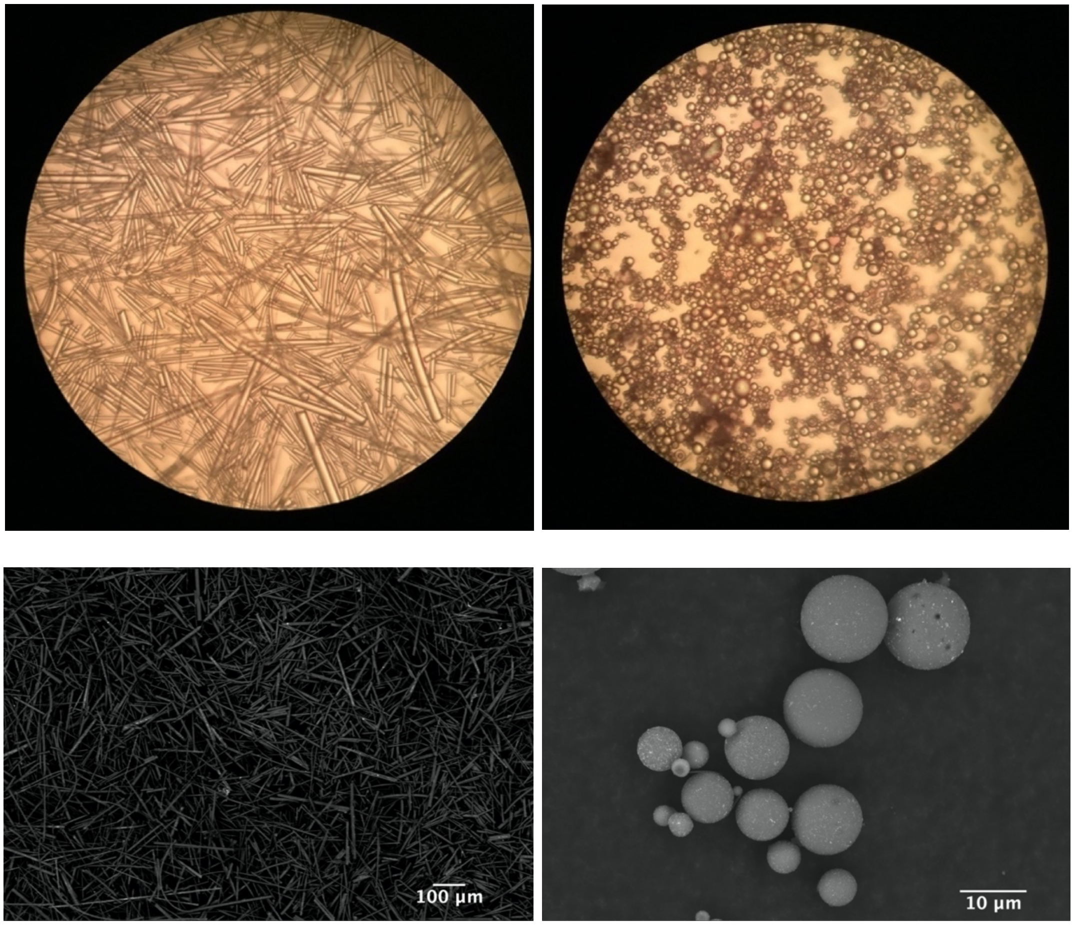

Figure S2. Optical images of polydisperse microfibers (top left) and glass microspheres (top right). Electron microscopy images (backscattered electrons) of the microfibers (bottom left) and glass microspheres (bottom right) showing the polydispersity of the substrates.

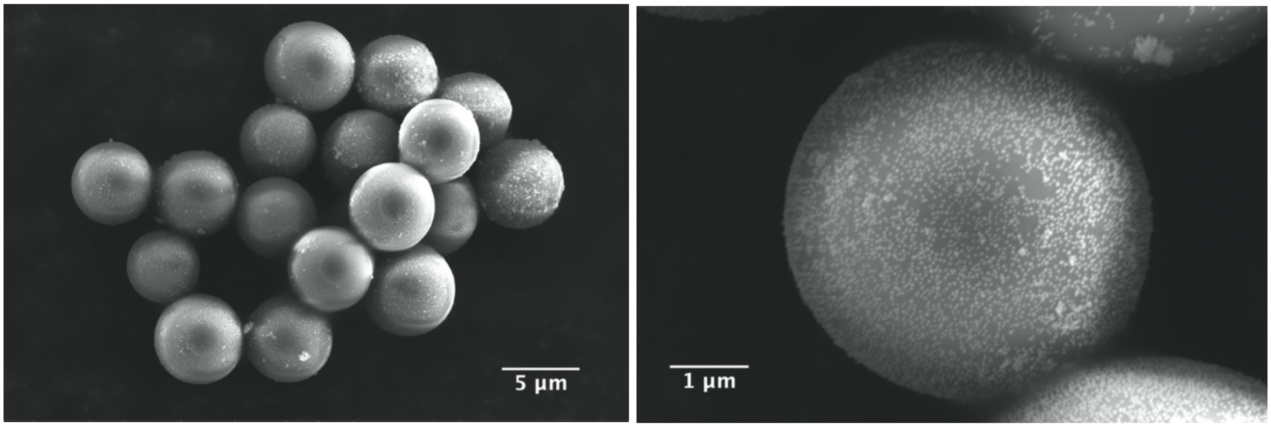

Figure S3. Characterization of the monodispersed glass microspheres of 5.3 μm. SEM images (secondary electrons) of the monodispersed 5.3 μm glass microspheres coated with Au NPs.

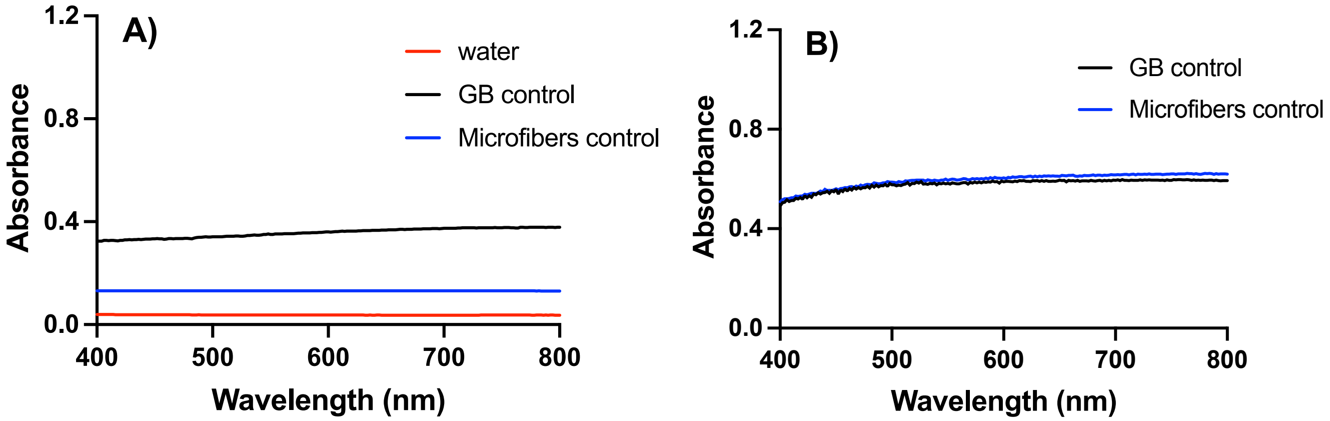

Figure S4. UV-Vis spectra for the bare substrates as an aqueous suspension (A) and as a dry film (B).

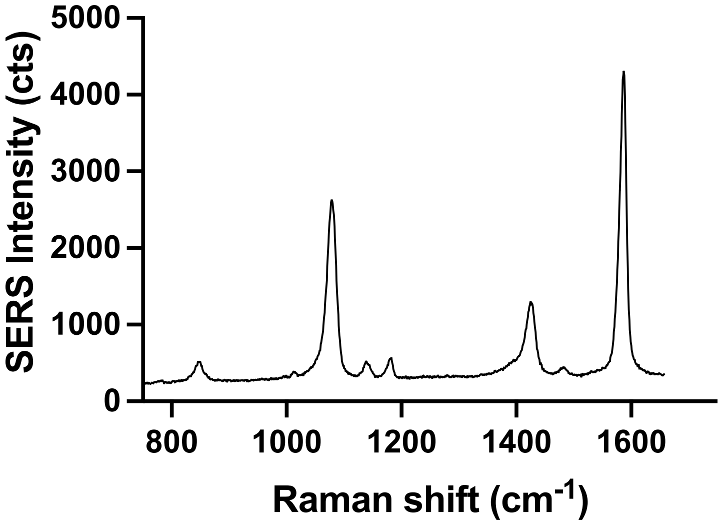

Figure S5. SERS spectra for 4-MBA. The peak at 1590 cm^{-1} was used to compare the difference in intensity with the various substrates.

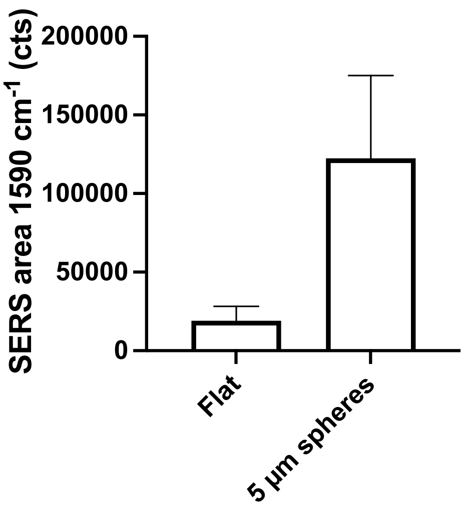

Figure S6. Comparison of the SERS response (4-MBA) for a flat substrate (n = 75) and the monodispersed glass microspheres of 5.3 µm (n = 75).

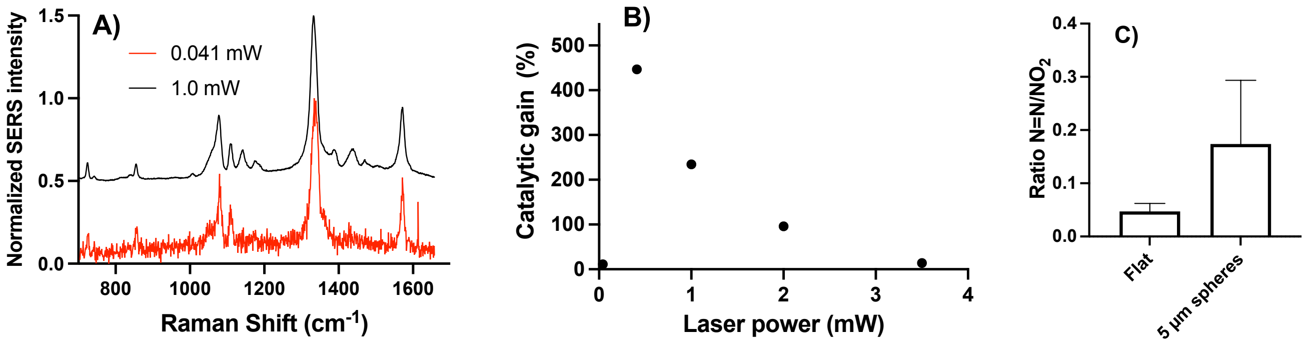

Figure S7. A) SERS spectra of unreacted 4-NBT obtained with a low laser power (0.041 mW) and of the product of the photocatalyzed reaction during irradiation at 1.0 mW. Both spectra are for a flat surface with a reaction time of 5 s. The relative intensity of the NO_2 peak (1342 cm^{-1}) decreases and new peaks associated to the diazo group (-N=N-) emerges (*e.g.* at 1437 cm^{-1}). B) Percentage increase in photocatalysis (catalytic gain) of with the glass microspheres in comparison to a flat substrate, both covered with Au NPs. C) Comparison of the initial photocatalysis after 5 s irradiation between the flat substrate (n = 75) and the monodispersed glass microspheres of 5.3 µm (n = 75).

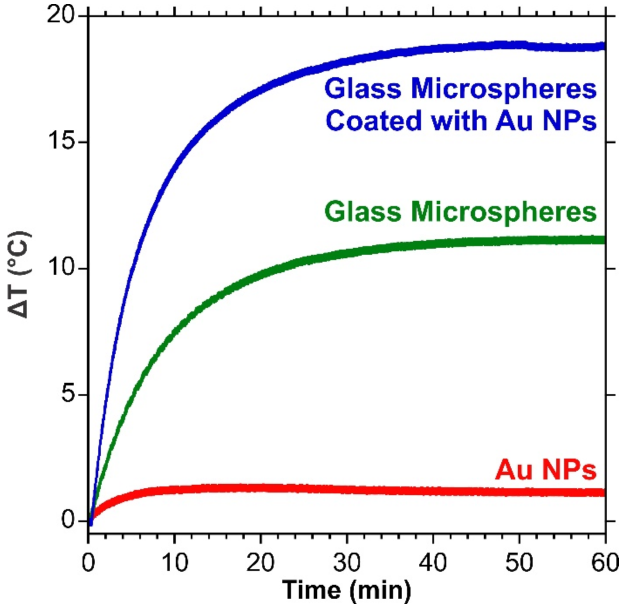

Figure S8. Photothermal heating of Au NPs on glass microspheres (blue) in comparison to bare glass microspheres (green) and a Au NP suspension (red).

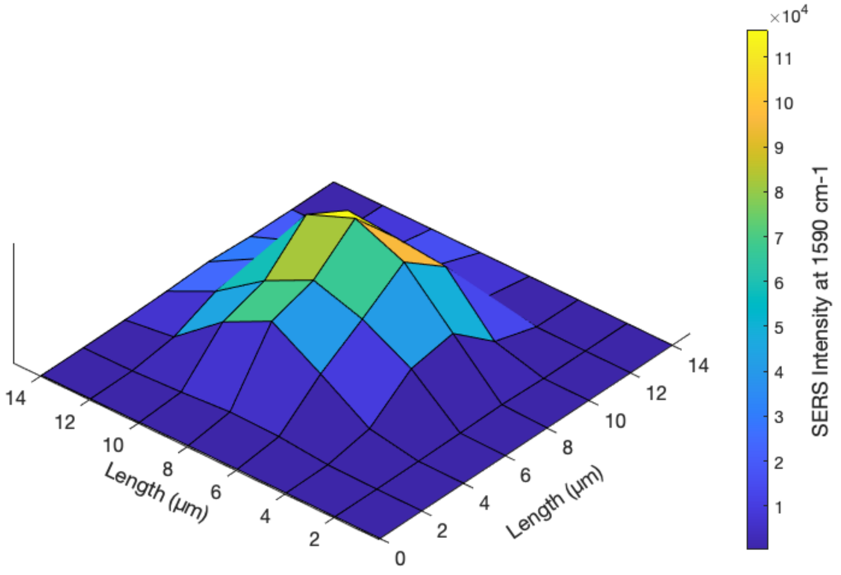

Figure S9. SERS image of the 1590 cm^{-1} peak of 4-MBA for a glass microsphere located at the center of the image. The maximum intensity coincides with the center of the microsphere. The color bar indicates the area of the SERS peak at 1590 cm^{-1}.

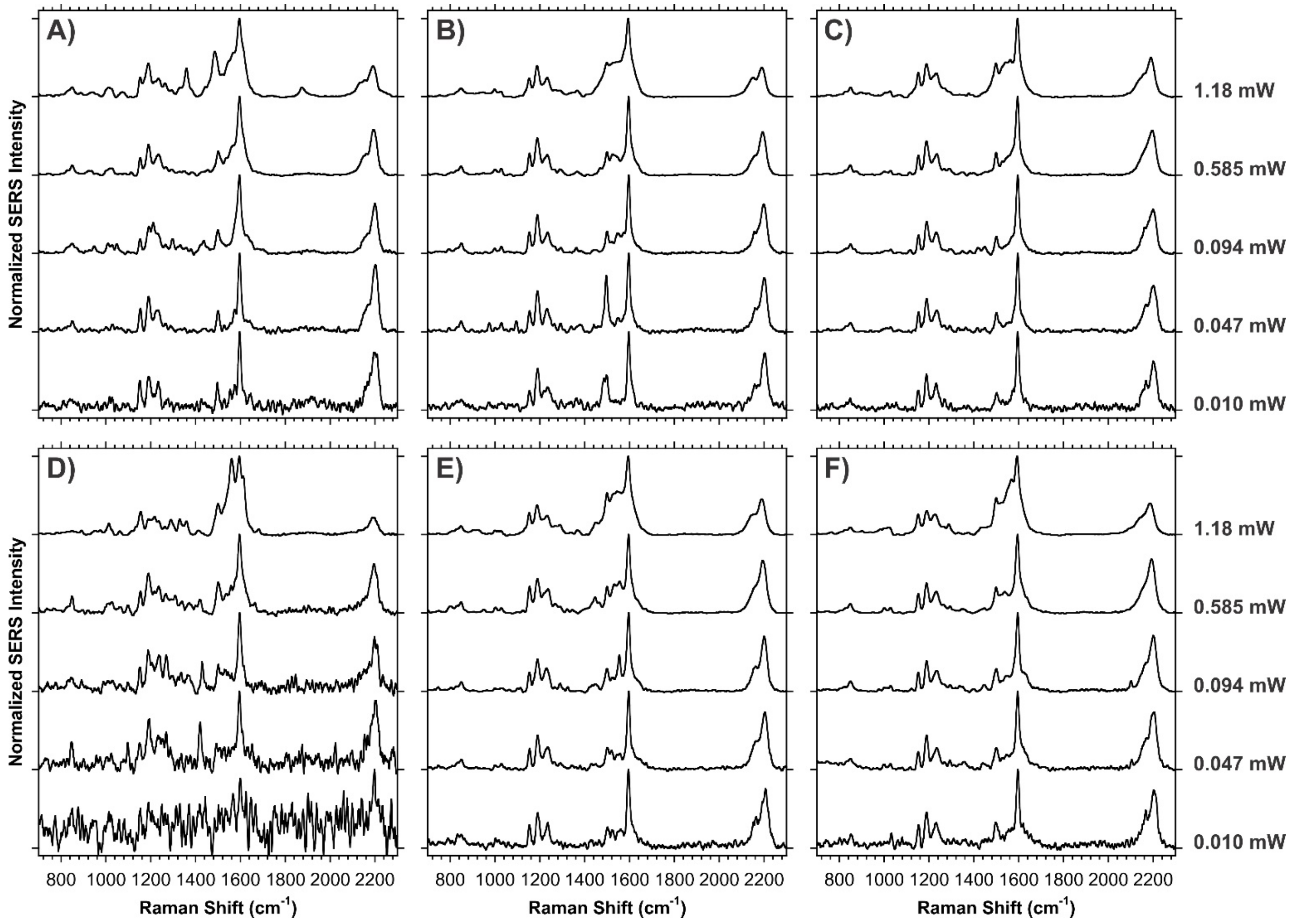

Figure S10. SERS spectra at different laser powers of 6 different Au NP coated glass microspheres functionalized with 4-fluorophenyl isocyanide (FPIC). The spectra are normalized and offset for clarity.

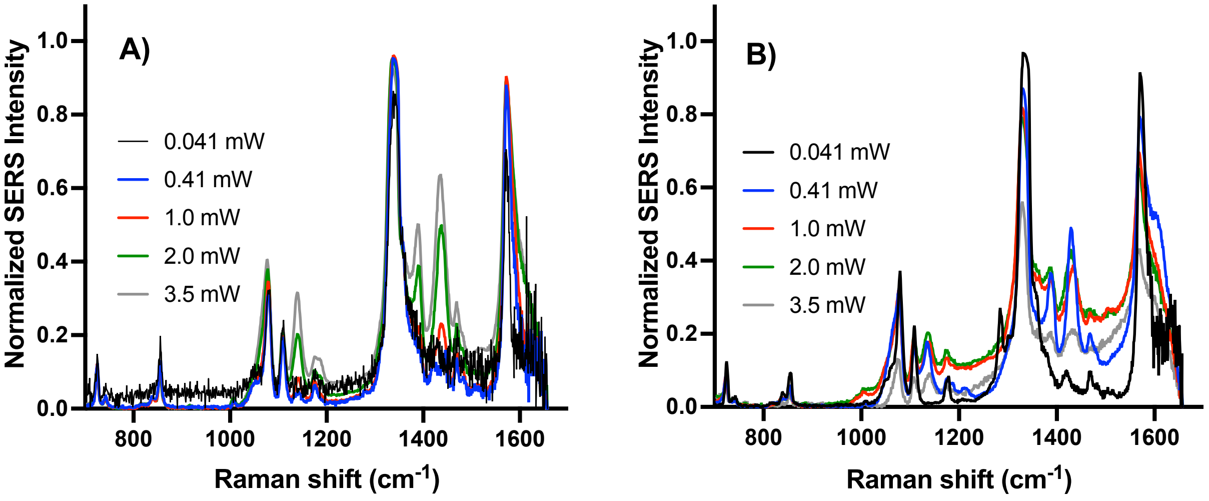

Figure S11. SERS spectra at different laser powers of Au NP coated on a flat substrate (A) or on glass microspheres (B) functionalized with 4-nitrobenzenethiol (4-NBT). The spectra are normalized for clarity.

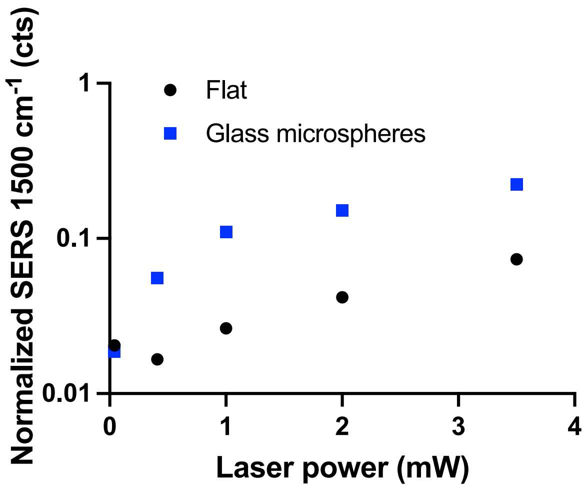

Figure S12. SERS intensity at different laser powers of Au NP coated glass microspheres and flat substrates functionalized with 4-nitrobenzenethiol (4-NBT). The background intensity at 1500 cm^{-1} measures the extent of carbonization (peak from 1400-1700 cm^{-1}).

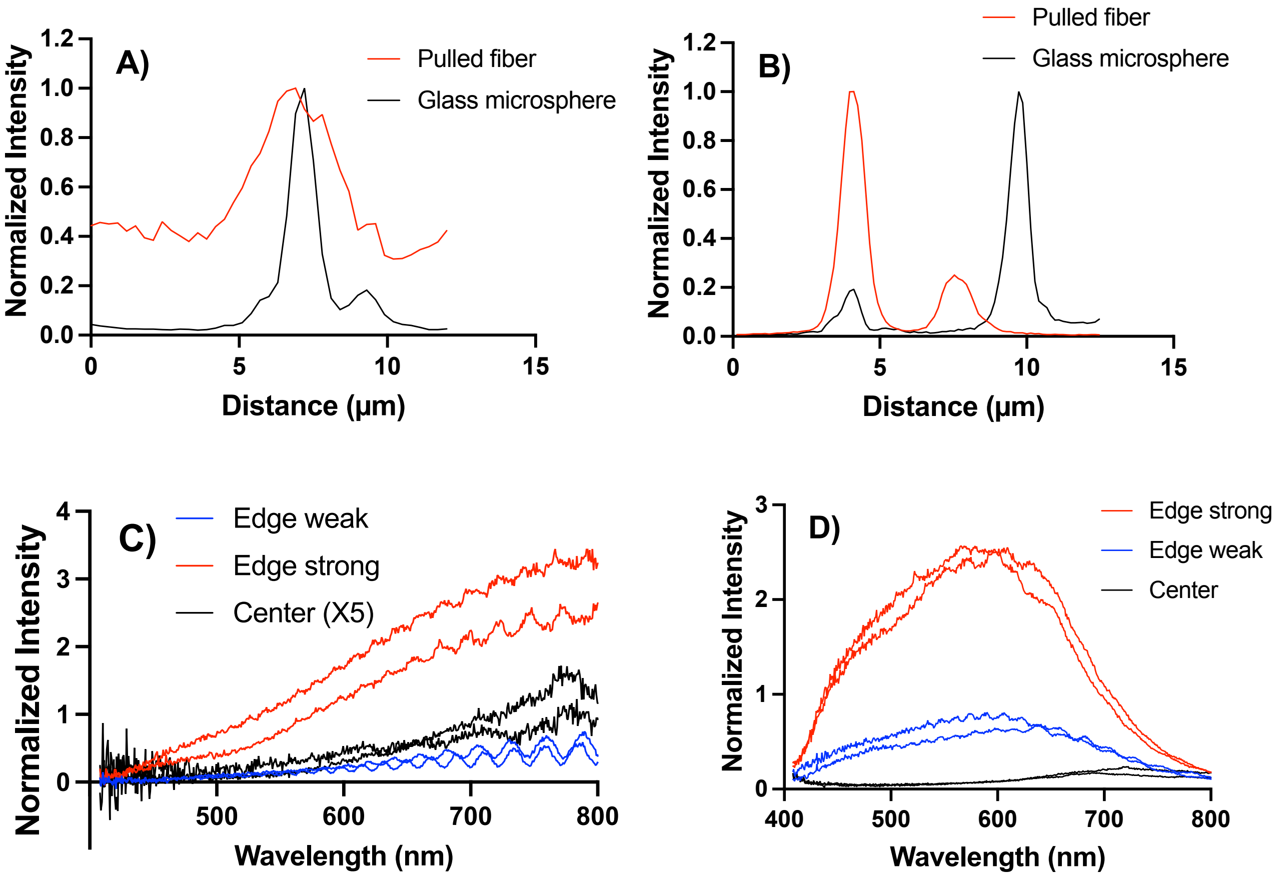

Figure S13. Cross-section of the light intensity for glass microspheres of approximately 5.3 µm and for a glass fiber with a similar diameter with collimated (A) and dark-field illumination (B). Dark-field optical spectra for different locations corresponding to the edge of the substrate with stronger light intensity, the center of the substrate and the opposite edge with a weaker light intensity on for glass microspheres of approximately 5.3 µm (C) and for a pulled fiber at a similar diameter (D). Waveguided optical resonances were observed from ~790 nm to ~620 nm and corresponds to modes with *l* between 31 to 39 in spherical substrates. These modes are also present in pulled fibers, albeit at lower intensity. It was also observed that light collected at the center of the microsphere did not exhibit resonances. Optical images of these substrates are provided in Figure 4.

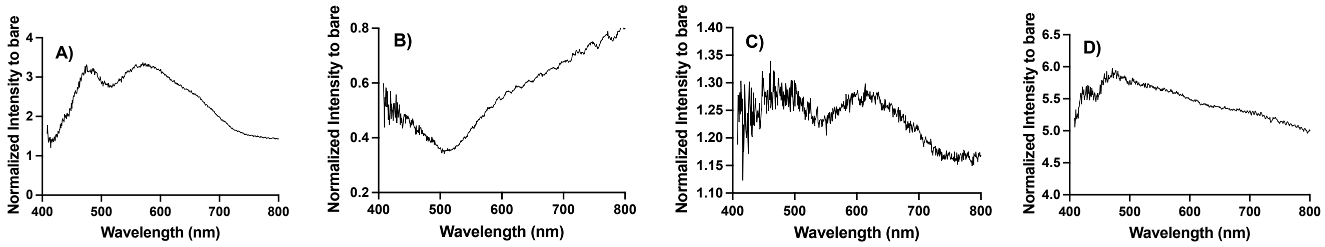

Figure S14. UV-Vis and dark-field scattering spectra for standard 5.3 µm glass microspheres and pulled fibers, for glass microspheres with collimated illumination (A), glass microspheres under dark-field illumination (B), pulled fiber under collimated illumination (C), and pulled fiber under dark-field illumination (D). Average for n = 8 for all UV-Vis and dark-field scattering spectra.

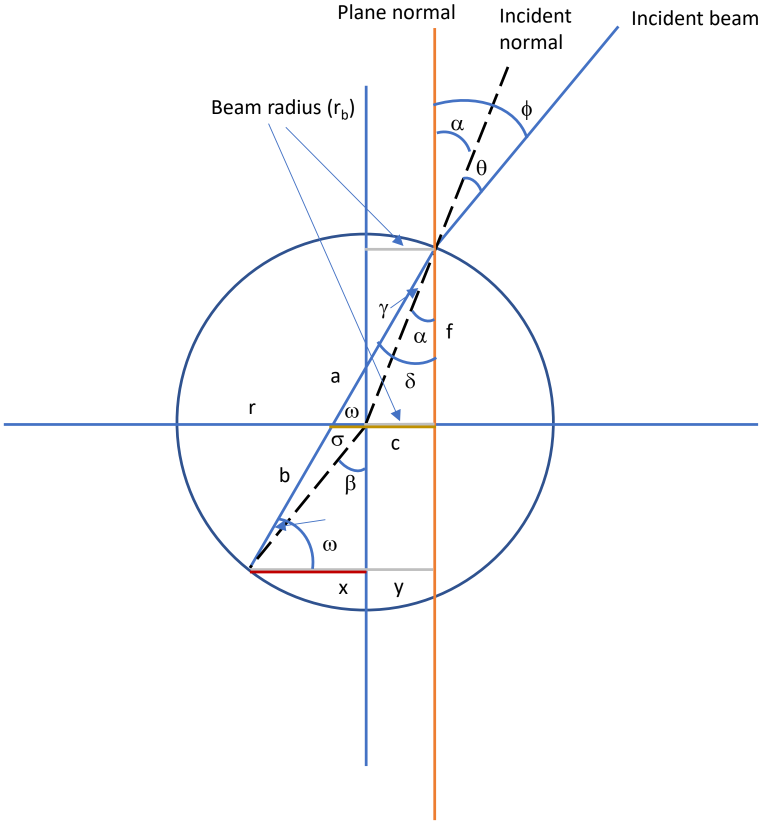

Figure S15. Scheme of the path focus rays and solid angle described at the opposite pole.

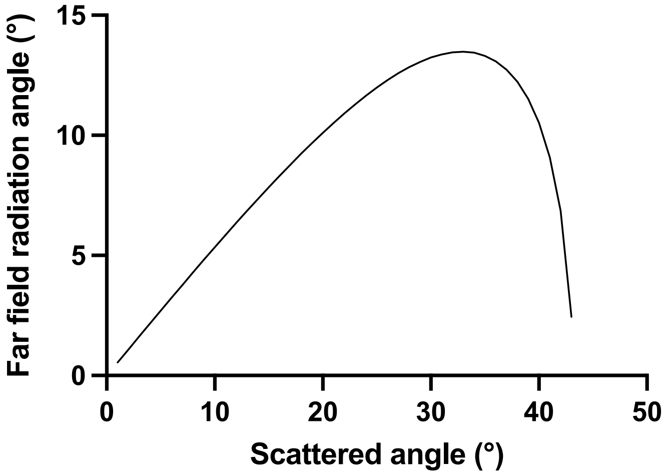

Figure S16. Far-field radiation angle as a function of scattered angle.

References:

1. https://www.edmundoptics.com/knowledge-center/application-notes/optics/understanding-ball-lenses/, accessed August 31st, 2022
